# Dynamics of SARS-CoV-2 immunity after vaccination and breakthrough infection in rituximab-treated rheumatoid arthritis patients: a prospective cohort study

**DOI:** 10.3389/fimmu.2024.1296273

**Published:** 2024-02-22

**Authors:** Hassen Kared, Ingrid Jyssum, Amin Alirezaylavasani, Ingrid M. Egner, Trung The Tran, Lisa Tietze, Katrine Persgård Lund, Anne Therese Tveter, Sella A. Provan, Hilde Ørbo, Espen A. Haavardsholm, John Torgils Vaage, Kristin Jørgensen, Silje Watterdal Syversen, Fridtjof Lund-Johansen, Guro Løvik Goll, Ludvig A. Munthe

**Affiliations:** ^1^ Department of Immunology, Oslo University Hospital, Oslo, Norway; ^2^ KG Jebsen Centre for B cell Malignancies, Institute of Clinical Medicine, University of Oslo, Oslo, Norway; ^3^ Precision Immunotherapy Alliance, Institute of Clinical Medicine, University of Oslo, Oslo, Norway; ^4^ Center for Treatment of Rheumatic and Musculoskeletal Diseases (REMEDY), Diakonhjemmet Hospital, Oslo, Norway; ^5^ Institute of Clinical Medicine, University of Oslo, Oslo, Norway; ^6^ ImmunoLingo Convergence Center, University of Oslo, Oslo, Norway; ^7^ Department of Gastroenterology, Akershus University Hospital, Lørenskog, Norway

**Keywords:** T cell, B cell, COVID-19, mRNA vaccination, rheumatoid arthritis, rituximab, breakthrough infection, ACPA

## Abstract

**Background:**

SARS-CoV-2 vaccination in rheumatoid arthritis (RA) patients treated with B cell-depleting drugs induced limited seroconversion but robust cellular response. We aimed to document specific T and B cell immunity in response to vaccine booster doses and breakthrough infection (BTI).

**Methods:**

We included 76 RA patients treated with rituximab who received up to four SARS-CoV-2 vaccine doses or three doses plus BTI, in addition to vaccinated healthy donors (HD) and control patients treated with tumor necrosis factor inhibitor (TNFi). We quantified anti-SARS-CoV-2 receptor-binding domain (RBD) Spike IgG, anti-nucleocapsid (NC) IgG, 92 circulating inflammatory proteins, Spike-binding B cells, and Spike-specific T cells along with comprehensive high-dimensional phenotyping and functional assays.

**Findings:**

The time since the last rituximab infusion, persistent inflammation, and age were associated with the anti-SARS-CoV-2 RBD IgG seroconversion. The vaccine-elicited serological response was accompanied by an incomplete induction of peripheral Spike-specific memory B cells but occurred independently of T cell responses. Vaccine- and BTI-elicited cellular immunity was similar between RA and HD *ex vivo* in terms of frequency or phenotype of Spike-specific cytotoxic T cells and *in vitro* in terms of the functionality and differentiation profile of Spike-specific T cells.

**Interpretation:**

SARS-CoV-2 vaccination in RA can induce persistent effector T-cell responses that are reactivated by BTI. Paused rituximab medication allowed serological responses after a booster dose (D4), especially in RA with lower inflammation, enabling efficient humoral and cellular immunity after BTI, and contributed overall to the development of potential durable immunity.

## Introduction

Rituximab, a CD20^+^ cell-depleting agent, has been proven efficient and safe for patients with rheumatoid arthritis (RA) (1, 2). As a consequence, rituximab is known to impair the humoral immunogenicity of influenza and pneumococcal vaccines (3). We (4) and others (5–8) have recently demonstrated that rituximab dramatically impairs serological SARS-CoV-2 vaccine responses after the basic administration of three doses (9–11). Moreover, we found that a third vaccine dose given within 6 months after a rituximab infusion failed to induce a serological response in most patients. Besides producing antibodies, B cells are important antigen-presenting cells for T cells as partners in concerted B and T cell immunity. However, we found that a third vaccine dose was necessary to provide T-cell responses in all patients (4).

If rituximab-treated RA patients lack protective antibodies, SARS-CoV-2-exposed individuals will rely on vaccine-expanded T cell responses to counteract the infection. The importance of T cells has been underlined in previous reports as T cells are necessary for the rapid and efficient resolution of COVID-19 (12, 13), for protection against severe COVID-19 in settings of low antibody levels (14), and for rapid viral control in the absence of antibodies—aborting infection in healthy individuals (15). T cell responses rely on appropriate T cell phenotypes, and correlates of protection have been found in COVID-19 patients (12, 13, 15, 16).

The phenotype and maturity of vaccine-generated B cell responses have not been described, and it is unknown if successful anti-receptor-binding domain (RBD) IgG responses in patients using rituximab wane with a decay rate that resembles that found in the healthy population or if rituximab-treated RA patients have an accelerated antibody decay rate as seen in other immunosuppressive/disease-modifying anti-rheumatic (DMARD) treatments (17–19).

The present study aimed to evaluate humoral and cellular immunity after vaccination and breakthrough infection (BTI) in rituximab-treated RA patients. We aimed to describe the induction and waning of anti-RBD IgG following four vaccine doses or three vaccine doses and BTI to identify clinical factors contributing to late seroconversion, to determine if patients could develop long-lasting Spike-specific memory B cells, and to deeply characterize the activation, differentiation, and exhaustion status of SARS-CoV-2-specific T cells, including immune responses to non-Spike antigens.

## Methods

### Study design and participants

Patients with rheumatoid arthritis who were on treatment with rituximab and followed in the Nor-vaC study (*Nor*wegian study of *vac*cine response to *C*OVID-19 vaccines in patients using immunosuppressive medication within rheumatology and gastroenterology) were included. Patients who were started on alternative medications after pausing rituximab were excluded from the analysis (see the **Supplementary Material** for details). In addition, patients in the Nor-vaC study treated with tumor necrosis factor inhibitors (TNFi) were included as patient controls for inflammatory rheumatic diseases. Patients were recruited from the Division of Rheumatology and Research at Diakonhjemmet Hospital in February 2021, before the initiation of the National Corona Vaccination program. The healthy donors (HD) were healthcare workers at Oslo University Hospital (OUS), Diakonhjemmet Hospital (DH), and Akershus University Hospital (AHUS). Nor-vaC is registered at clinicaltrials.gov (NCT04798625) and has ethics approval (Regional Committees for Medical Research Ethics Southeast Norway, reference numbers 235424, 135924, and 204104). All participants provided written informed consent.

### Procedures

SARS-CoV-2 vaccines were provided to patients and HD by the Norwegian Corona Vaccination Program, with three doses plus one booster in patients and two doses plus one booster in HD. The patients included in the Nor-vaC study with low levels of neutralizing IgG anti-RBD levels (4) were recruited into a separate intervention study (EudraCT Number: 2021-003618-37) and provided a third vaccine dose in July–August 2021. The remaining patients received the third dose from the Norwegian Corona Vaccination program later in 2021. Dose (D) 4 was a booster dose and was provided in December 2021. Patients with COVID-19 after the initial vaccine series of three doses were not eligible for a booster dose. According to the national vaccination program, boosters were administered as either a half-dose of mRNA-1273 or as a full dose of BNT162b2.

Serum samples donated 2–4 weeks and 3 and 6 months after D2, D3, D4 and breakthrough infections (BTI) were analyzed for IgG antibodies to the full-length Spike protein from SARS-CoV-2, RBD, and anti-nucleocapsid antibodies. The seroconversion was defined as >2,000 BAU/mL and 5 AU/mL, respectively (4, 20). Breakthrough infections were self-reported by response to a monthly questionnaire (after a positive PCR test or lateral flow test) or by the development of anti-nucleocapsid antibodies [see the **Supplementary Methods** for the measurement of antibodies against citrullinated proteins (ACPA), inflammatory markers with ELISA, and Olink Target 96 inflammatory panel]. Thawed peripheral blood mononuclear cells (PBMCs) were tested for functional T cell responses or stained with antibody panels [see **Supplementary Methods** and (4, 21)] to define the phenotype of specific T cell responses over unstimulated background and HLA-restricted Spike-specific CD8 T cells and B cell responses to RBD or Spike protein (21).

### Outcomes

The outcomes of this study were the induction and decay of humoral response assessed by anti-RBD IgG levels, anti-nucleocapsid response after BTI in vaccinated patients with different inflammatory statuses, and the activation, differentiation, and functional status of B and T cell responses to Spike peptides following SARS-CoV-2 vaccination and BTI.

### Statistical analysis

A comparison between paired samples in patients and controls was performed by Wilcoxon matched-pairs signed-rank test and with Mann–Whitney *U*-test. All tests were two-tailed and conducted at the 0.05 significance level. Analyses were carried out using GraphPad Prism version 9. High-dimensional phenotypic profiles and sample distributions were shown using uniform manifold approximation and projection. Data analysis was performed using CYTOGRAPHER^®^ (ImmunoScape cloud-based analytical software), custom R-scripts, GraphPad Prism (GraphPad Software), and FlowJo v10 software (BD Life Sciences). Statistical significance was set at a threshold of * for *p* < 0.05, ** for *p* < 0.01, and *** for *p* < 0.001.

### Role of the funding source

The study was funded by the Coalition for Epidemic Preparedness Innovations (CEPI), a KG Jebsen Foundation (grant 19), Oslo University Hospital, University of Oslo, the South-Eastern Norway Regional Health Authority, REMEDY, The Centres for Clinical Treatment Research (FKB), and The Research Council of Norway (project number 328657). The funders of the study had no role in the study design, data collection, data analysis, data interpretation, writing of the report, or the decision to submit the paper for publication.

## Results

A total of 76 patients provided blood samples (72 after D2, 70 after D3, 61 after D4, and 5 after D3 and BTI) and were eligible for inclusion (**Table 1**). The patients were treated with rituximab in monotherapy [19/76 (26%)] or in combination with a csDMARD [59/76 (75%)], mostly methotrexate (**Table 1**). A total of 82 patient controls treated with TNFi and 168 HD were included (**Table 1**). Due to the pandemic and vaccination regimen, rituximab administration was delayed or discontinued in many patients. The last administration of rituximab was before D1 for 25/76 patients [33%] and after D3 or D4 for 15/76 patients [20%]. The workflow is summarized in **Supplementary Figure S1A**.

**Table 1:** Patients’ characteristics.

	All rituximab^a^ *N* = 76	Rituximab for cellular analyses *N* = 30	TNFi, serology *N* = 82
**Age, median (IQR)**	60.0 (54.5–67.0)	60.0 (54.0–67.0)	48 (40–58)
**Female sex, no. (%)**	64 (84)	26 (87)	40 (49)
**Rituximab monotherapy, no. (%)**	18 (24)	8 (27)	–
**Comedication** Methotrexate^b^ Prednisolone^c^ Sulfasalazine Leflunomide Plaquenil	32 (42) 12 (16) 4 (5) 9 (12) 1 (1)	15 (50) 1 (3) 2 (7) 3 (10) 1 (3)	– – – – –
**Time on rituximab treatment before D1, median years (IQR)**	6.0 (2.9–8.9)	4.8 (2.7–9.2)	–
**Number of rituximab infusions before D1, median (IQR)**	10.5 (4.5–16.0)	9.0 (5.0–18.0)	–
Time from last rituximab infusion to vaccine or BTI, days (IQR)			
Dose 2 Dose 3 Dose 4 BTI	171 (125–214) 187 (132–226) 289 (166–369) 267 (140–432)	150 (121–239) 180 (124–226) 284 (144–363) 212 (126–370)	– – – –
Vaccine types, *n* (%)^d^			
**Doses 1 and 2** mRNA-1273 BNT162b2	17 (22) 59 (78)	9 (30) 21 (70)	18 (22) 63 (78)
**Dose 3** mRNA-1273 BNT162b2 CBA01	24 (32) 49 (66) 1 (1)	11 (37) 19 (63) 0	41 (51) 40 (49) 0
**Dose 4** mRNA-1273 BNT162b2 CBA01 CBA45	32 (47) 30 (45) 3 (5) 2 (3)	13 (48) 12 (44) 1 (4) 1 (4)	25 (46) 27 (50) 2 (4) 0
**BTI the whole period, *n* (%)**	51 (67)	21 (70)	45 (55)
**Time from last vaccine to BTI, median days (IQR)**	103 (55–169)	100 (48–169)	95 (45–136)
**Number of vaccines before BTI, mean (SD)**	3.6 (0.6)	3.7 (0.5)	–
**BAU after the last vaccine before BTI, median (IQR)**	702 (4–2,315)	1,483 (76–3,998)	–

BTI, breakthrough infection; D1, vaccine dose 1; IQR, interquartile range.

a A total of 76 were included and 30 of these also in cellular analyses.

b Median-dose methotrexate per week: 14 mg (IQR, 10.0–20.0).

c Median dose of prednisolone per day: 5 mg (IQR, 5.0–6.3).

d Missing in one TNFi patient.

### Improvement of vaccine responsiveness after a boosting dose in RA

**Figure 1:**
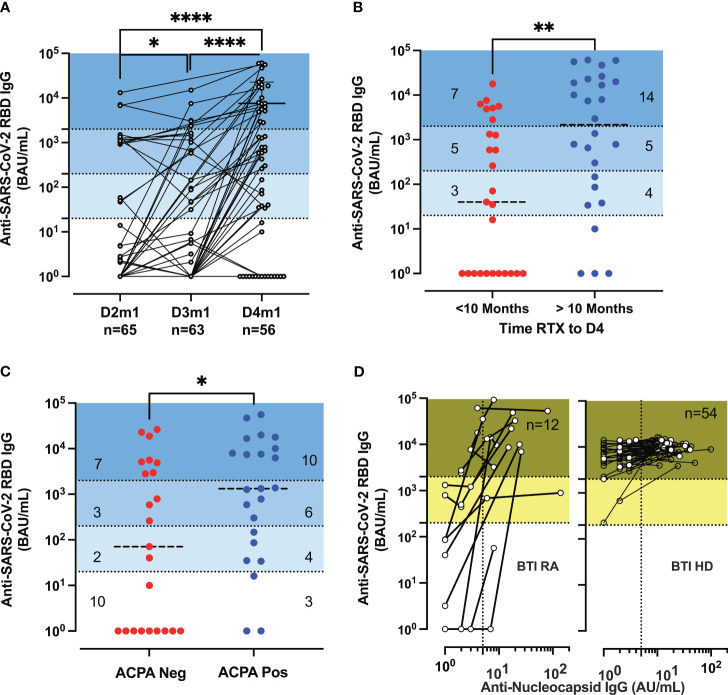
Serological responses in rituximab-treated rheumatoid arthritis (RA) patients. **(A)** Serological response post-vaccination. Longitudinal IgG anti-RBD (BAU/mL) 1 month (m1) after dose 2 (D2) to dose 4 (D4) in patients (n = 65, 63, 55) with at least two samples. Median titer for RA D4 was 586 BAU/mL [IQR: 1–7,390]. Wilcoxon matched-pairs signed-rank test; P-values are indicated. The P-values are indicated as *for p < 0.05 and ****p < 0.0001. **(B)** Serological response post-D4 of vaccine and rituximab therapy. IgG anti-RBD (BAU/mL) responses after D4 of the vaccine according to the time interval since the last rituximab infusion with a median of 288 days [IQR: 155–361] corresponding to 10 months (within the last 10 months in red or more than 10 months in blue) before vaccination. IQR of 74–20,726 BAU/mL for RA with more than 10 months and IQR of 1–2,814 BAU/mL for RA with recent therapy. **(C)** Serological response post-vaccination and anti-citrullinated protein auto-antibodies (ACPA). IgG anti-RBD (BAU/mL) in ACPA-negative [IQR: 1–4,911] and ACPA-positive RA [IQR: 86–10,079] were measured at D4. **(D)** Serological responses before and after breakthrough infections. The biplots show IgG anti-RBD (BAU/mL) vs. IgG anti-nucleocapsid (AU/mL) in patients and healthy donors (HD); the samples are sequential after dose 3 (D3) or D4. The IgG anti-nucleocapsid titer has an IQR of 7.5–22 and n = 12 for breakthrough infection (BTI) RA and an IQR of 10.7–22.5 and n = 54 for BTI HD. The P-values are indicated as *for p < 0.05, **for p < 0.01, and ****for p < 0.0001 (see also **Supplementary Figure S1** for further analysis of serological responses and decay of specific IgG).

First, we analyzed the induction and the stability of the humoral response after doses 3 and 4 in RA. The serology analyses included 65 patients, compared to 168 HD. **Figure 1A** shows the longitudinal humoral responses after each vaccine dose in patients that had not been infected with SARS-CoV-2 (no SARS-CoV-2 PCR-positive tests nor had developed anti-nucleocapsid responses). The level of anti-RBD IgG increased gradually in patients receiving a third and fourth dose (*p* < 0.047: D2 vs. D3, *p* < 0.0001: D3 vs. D4) (**Figure 1A**). A total of 35% (20/56) were over the indicative level for high responders at 2,000 BAU/mL after D4 [vs. 4.6% (3/65) at D2 and 9.5% (6/63) at D3] (**Figure 1A**). However, the responses remained significantly lower than in HD after D3 (*p* < 0.0001 for RA D3 and *p* = 0.0007 and for RA D4 in patients vs. HD D3), with the indicated number of patients classified as non-responder (<20 BAU/mL), low-responder (20–200 BAU/mL), middle responder (200–2,000 BAU/mL), and high responder (>2,000 BAU/mL) (**Supplementary Figure S1B**).

Patients treated with rituximab in monotherapy or on concomitant csDMARD treatment did not differ significantly regarding the serological responsiveness to the SARS-CoV-2 vaccination. Next, we sought to determine if the level of antibody response was associated with the interval time between D4 and the last administration of rituximab (median of 288 days). A positive correlation was established between the interval time and the anti-SARS-CoV-2 RBD IgG titer (*r* = 0.5433, *p* < 0.0001) (**Supplementary Figure S1C**). Patients with more than 288 days (10 months) since the last rituximab infusion had a higher titer (median titer: 2,169 BAU/mL) than patients with recent therapy (median titer: 40 BAU/mL; *p* = 0.0033) (**Figure 1B**). Finally, we investigated whether the interval time since the last rituximab infusion could affect the markers of immunopathogenesis in RA (measured at D4 by the detection of antibodies against citrullinated proteins, ACPA) and thus identify indirectly patients more prone to generate vaccine-elicited antibodies (**Figure 1C**). We completed the disease activity after D2 (4) with the analysis of patients after D4. ACPA-positive patients possessed a higher titer of anti-SARS-CoV-2 RBD IgG (median level: 1,331 BAU/mL vs. median level: 71 BAU/mL; *p* = 0.043). To assess the isotype switching after vaccination, we measured the total and IgG subclass titer (IgG_1,2,3,4_). The level of total IgG was in the range of values detected in HDs and was increased for ACPA-positive patients (median level: 7.45 g/L vs. median level: 6.19 g/L) (*p* = 0.012), suggesting a resurgence of B cells due to the paused rituximab infusion (**Supplementary Figure S1D**).

The waning of humoral immunity is a crucial issue for the long-term protection of immune-suppressed patients. We detailed the decay rate within 3 months after D2, D3, and D4 for responder RA (>1,000 BAU/mL). The results were different after D3 between HD and RA (*p* < 0.0001) but similar between D3 HD and D4 RA (*p* = 0.20) but were lower than those found in patients treated with TNFi (*p* < 0.0001) (**Supplementary Figure S1E**).

BTI increased the IgG anti-Spike responses and induced the development of IgG anti-nucleocapsid (NC) (median 14 AU/mL, *n* = 12) but did not significantly differ from that found in HD with BTI (median 14 AU/mL, *n* = 54) (**Figure 1D**).

### Persistent inflammation after seroconversion in RA

First, we sought to delineate whether ongoing systemic inflammation could interfere with the vaccine-elicited immune response. RA (*n* = 11) had signs of inflammation compared to HD before D1 with a significantly higher GDF-15 (median 944 pg/mL, *p* < 0.0001), calprotectin (median 33600 pg/mL, *p* = 0.012), and IL-6 (median 14.2 pg/mL, *p* = 0.032) but not for C3a, CXCL4, Galectin-9, IP-10, LBP, MPO, sCD163 and Zonulin (**Supplementary Figures S2A, B**). We established an inflammatory atlas of RA patients after D4 or BTI in **Supplementary Figure S2B**. The patients were stratified according to vaccine responsiveness and clinical parameters (age, therapy, and auto-immune antibodies). The quantification of proteomics depicted a persistent inflammation even in high-responder RA after D4 (**Supplementary Figure S2C**). The expression of the proteins was visualized by PCA, enabling the identification of vaccine-specific signatures in HD and RA patients. If the BTI signatures were different in RA and HD, it was more difficult to distinguish RA patients after vaccination and natural infection (**Supplementary Figure S2D**). We quantified the fold change and the significance between these different groups (**Supplementary Figure S2E**). To limit the consequences of individual analyte variation and stratify inflammation, we defined an inflammatory score (IS). First, the difference between the groups was quantified (*p* = 0.0013 and *p* = 0.0067 for vaccines and BTI, respectively) and revealed in RA D4 two sub-groups of patients (**Supplementary Figure S2F**). Next, we sought to investigate whether clinical parameters can explain this difference. Prolonging the interval time between rituximab infusion and vaccination was the most significant factor (median time of 131 vs. 338.5 days for IS high and low, respectively; *p* = 0.0006). Age also contributed to the inflammatory score (*p* = 0.039). Finally, we observed that serology-high-responder RA presented less inflammation than vaccine-non-responder RA (*p* = 0.020). As mentioned in **Figure 1C**, the response to the vaccine was associated with ACPA and total IgG concentration. We were able to establish a positive correlation between IL-12p40 (cytokine driving Th1 and Th17 response) and ACPA titer in D4 RA patients. Our results were confirmed by *in silico* pathway analysis and the specific enrichment for rheumatoid arthritis as well as the IL-17 signaling pathway (**Supplementary Figure S2H**).

### Memory B cell response after the booster vaccine dose and BTI

Long-lasting humoral immunity requires the persistence of isotype-switched antibodies and the differentiation of long-lived plasma cells from germinal center B cells. A high-dimensional analysis of B cells was performed to identify the cellular source of anti-SARS-CoV-2 RBD IgG detected in serological-responder RA after vaccination or BTI. The B cell responses were analyzed in 6, 15, 9, and 5 RA after D2, D3, D4, and BTI, respectively (**Supplementary Table S1**).

**Figure 2:**
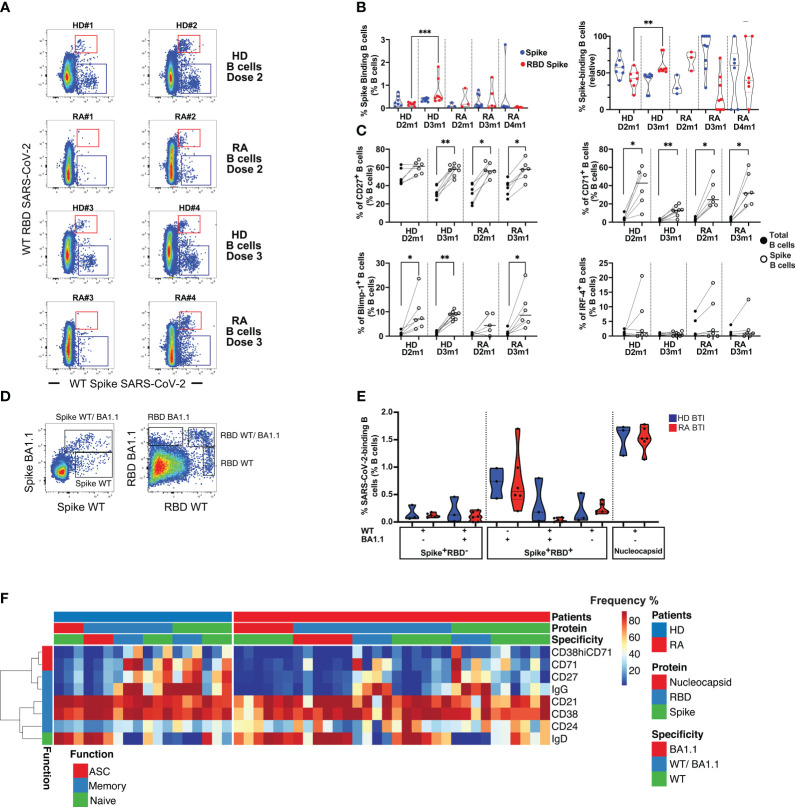
Humoral response in rituximab-treated rheumatoid arthritis (RA) patients after vaccination and infection. **(A)** Identification of Spike- and RBD-binding B cells. The representative dot plots show B cells that bind both wild-type (WT) Spike and WT RBD (upper region, Spike^+^ RBD^+^) or WT Spike but not WT RBD (lower region, Spike^+^ RBD^-^). Examples are shown for two serological responder RA and two healthy donors (HD) 1 month after D2 and D3. **(B)** Quantification of Spike^+^ RBD^+^ vs. Spike^+^ RBD^–^binding B cells. Left: frequency of Spike-binding B cells Spike^+^ RBD^+^ or Spike^+^ RBD^-^ 1 month after the vaccine in HD (D2-3) vs. rituximab-treated patients (D2-4). Right: relative distribution of Spike^+^ RBD^+^ vs. Spike^+^ RBD^-^ binding B cells 1 month after vaccination in HD (D2-D3) and RA patients (D2-D4). Mann–Whitney test; two-tailed *P*-values <0.01 (**) and <0.001 (***). **(C)** Phenotype of Spike-binding B cells. Frequency of Spike-binding and total B cells expressing CD27, CD71, Blimp-1, or IRF-4 1 month after D2 and D3. Wilcoxon matched-pairs signed-rank test, *P*-value with * for *p* < 0.05 and ** for *p* < 0.01. **(D)** WT or Omicron Spike- and RBD-binding B cells after breakthrough infection (BTI). Left panel: detection of cross-reactive CD19^+^ B cells that bind both Omicron (BA.1.1) Spike and WT Spike, upper region vs. only WT Spike (lower region, WT Spike^+^ BA.1.1 Spike^-^). Right panel: detection of CD19^+^ B cells that bind both Omicron and WT RBD (WT RBD^+^ BA1.1^+^), Omicron RBD only (WT RBD^-^ BA1.1^+^), or WT RBD only (WT RBD^+^ BA1.1^-^). **(E)** Quantification of WT or Omicron Spike- and RBD-binding B cells after BTI. Distribution of Spike and RBD-binding B cells as shown in **(D)**. For HD and RA, left, IQR [0.091%–0.16%] for WT Spike, IQR [0%–0.22%] for cross-reactive WT/BA.1.1 Spike; IQR [0.17%–0.34%] for RBD WT only, IQR [0%–0.074%] for RBD WT/BA.1.1, and IQR [0.41%–1.2%] for RBD BA1.1-only. Percentage of nucleocapsid-binding B cells, right, with IQR [1.4%–1.7%]. Mann–Whitney test; *P*-value non-significant. **(F)** Phenotype of SARS-CoV-2-specific B cells after BTI. Spike- and RBD-binding B cells with specificities as in **(D)**. For Omicron BA1.1-only, cross-reactive (WT/BA.1.1) and WT-only Spike/RBD, or nucleocapsid. The heat plots show differentiation markers as indicated, clustered into a function: antibody-secreting cells (ASC), memory, and naïve (see also **Supplementary Figure S3** for an in-depth analysis of B cell phenotypes and specificities).

Overall, rituximab-treated patients had very few peripheral B cells (data not shown). These few B cells were immature, activated (CD38), and expressed IgD and IgM. Vaccine responder patients (>200 BAU/mL), however, had some reconstituted B cells, expressing light-chain Ig kappa and Lambda with an activated profile (CD71, HLA-DR in clusters 1 and 2) (**Supplementary Figures S3A–D**). Among responder patients after D2 or D3, we were able to detect the low frequency of Spike-binding B cells that either bound the receptor-binding domain (RBD) of Spike (Spike^+^RBD^+^) or that could bind Spike but not RBD (Spike^+^RBD^-^) [**Figure 2A**; the regions were as previously described (21, 22)]. At 1 month after D2 (D2m1) and D3 (D3m1), 3/6 and 5/6 responder patients had both RBD^+^ Spike^+^ B cells and RBD^-^ Spike^+^ B cells (**Figure 2B**). The Spike-binding B cells were significantly enriched in CD27^+^ memory cells (*p* = 0.0078 and *p* = 0.031 for HD and patients, respectively), the CD71 activation marker (*p* = 0.031, D2m1 and *p* = 0.0078, D3m1 for HD and *p* = 0.031, D2m1 and *p* = 0.031, D3m1 for RA, respectively), and low-level expression of Blimp-1 transcription factor (significant only after D3 in patients, *p* = 0.031). The emergent vaccine immunity consisted of activated memory B cells (CD27^+^CD71^+^Blimp-1^+^) that did not significantly express the plasmablast marker IRF-4 (**Figure 2C**).

We sought to determine whether the vaccine responsiveness after D4 was coupled with an expansion and/or recovery of peripheral B cells. A strong positive correlation was established between the frequency of total B cells (CD19^+^) and anti-RBD IgG after D4 (**Supplementary Figure S3E**). However, we still observed unresponsive patients and persistent rituximab effects. Despite the exclusion of patients with a limited number of B cells, we detected only low frequencies of peripheral SARS-CoV-2 Spike-binding B cells after D4 in responder patients (**Figure 2B**). Most of the memory B cell responses were directed against Spike after D3 and D4 in patients, while RBD-binders dominated in HD after D3.

Next, the patients were analyzed after BTI to evaluate if natural infection might enhance the number and functionality of Spike-binding B cells as suggested by serological observations (**Figure 1D**). Since these patients were infected by Omicron (BA.1.1) VOC, we analyzed if B cells could bind wild-type (WT) Spike as well as mutated BA.1.1 Spike and wild-type RBD and mutated BA.1.1 RBD (**Figure 2D**; **Supplementary Figure S3F**). This analysis revealed B cells that (1) could bind only WT Spike/RBD and not BA1.1, (2) B cells that were cross-reactive and bound both WT and BA1.1 Spike/RBD (both vaccine-related B cell subsets), and (3) B cells that bound only BA.1.1 Spike/RBD (B cells that did not bind to vaccine Spike). After BTI, the patients had normalized levels of B cells that could bind all combinations of probes similarly to HD (median 0.11% for WT Spike, median 0.093% for cross-reactive WT/BA.1.1 Spike, median 0.2% for RBD WT only, median 0.024% for RBD WT/BA.1.1, and median 0.56% for RBD BA1.1 only) (**Figure 2E**). Moreover, RA BTI and HD BTI had similar frequencies of B cells that bound nucleocapsid (median 1.5%), a finding that demonstrated *de novo* B cell responses toward Spike/RBD epitopes not found in the vaccine and nucleocapsid responses corresponding to the anti-NC IgG responses described above.

Next, we assessed the isotype switching (to IgG), activation (CD38 and CD71), and differentiation (CD21, CD24, and CD27) status of antigen-specific B cells after BTI. In-depth phenotyping of RBD-, Spike-, and nucleocapsid-binding B cells after BTI demonstrated that the expanded Spike-binding B cells still had a less mature phenotype even after BTI. However, the vaccine-related cross-reactive B cells that could bind both WT as well as Omicron had isotype switched to IgG. *De novo* responses, i.e., B cells that bound nucleocapsid or Omicron-only Spike/RBD were IgD^+^, while WT Spike/RBD was intermediate in phenotype (IgD/IgG and expressed differentiation markers) (**Figure 2F**).

### Induction of Spike T cell responses after the booster vaccine dose and BTI

We sought to evaluate whether the chronic condition (inflammation, drug toxicity, and alteration of B cells) of patients in our cohort might dampen the induction of a persistent and protective cellular immune against SARS-CoV-2 and its variants. T-cell responses were analyzed in 20, 29, 9, and 5 RA after D2, D3, D4, and BTI, respectively (**Supplementary Table S1**). First, an in-depth analysis of cellular phenotypes and subset distribution was performed before and after standard SARS-CoV-2 vaccination. The unsupervised analysis of mass cytometry experiments revealed the emergence of CD8 T cells expressing activation markers (CD71^+^HLA-DR^+^PD-1^+^CD95^+^) but not exhaustion markers (CD160^-^TIM-3^-^KLRG1^Low^) (cluster 16). Late-differentiated memory CD4 T cells were elicited by vaccination and were characterized by the expression of KLRG1 and CD57 (cluster 10), suggesting a positive T cell vaccine response with the appropriate effector phenotype (21, 23) (**Supplementary Figures S4A–C**). Further validation by flow cytometry analysis (supervised) revealed a significant shift from naïve to effector for CD4 and CD8 T cells as well as reduced CD127 and CD27 expression of CD8 T cells, suggesting functional maturation induced by the vaccination (**Supplementary Figures S4D, E**). This in-depth analysis revealed that vaccination (two doses) has modulated the subset distribution. We extended this analysis to Spike-specific T cells.

**Figure 3:**
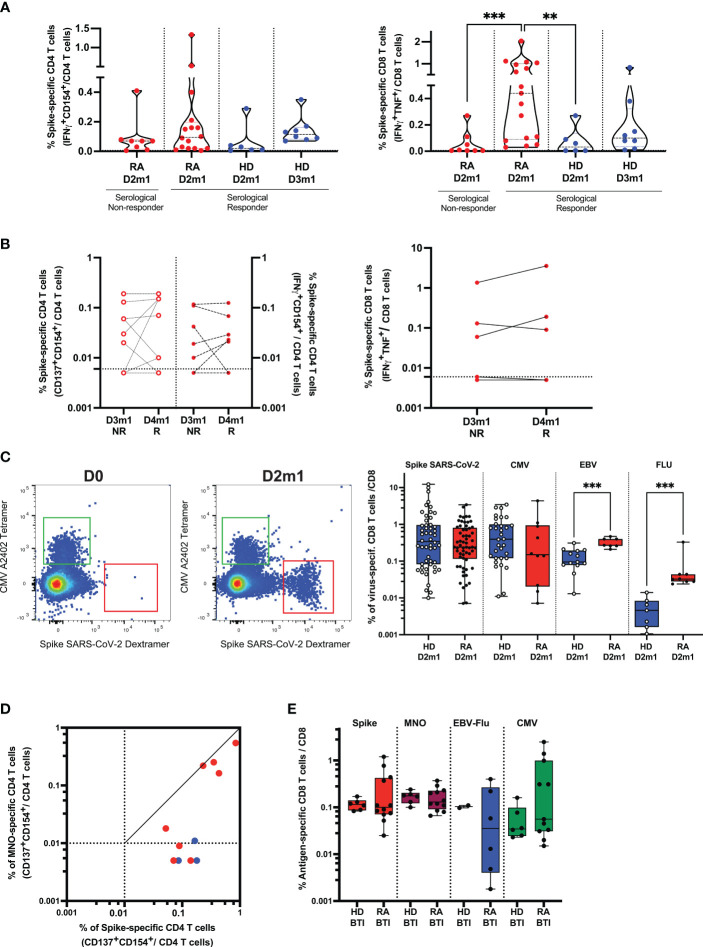
Cellular response in rituximab RA patients after vaccination and infection. **(A)** Functionality of SARS-CoV-2 specific T cells and serological response. Frequency of Spike-specific T cells after D2 in serological responder and non-responder RA. The frequency of response from unstimulated cells was subtracted as a non-specific background [see **Supplementary Methods** and (4, 21)]. Gated CD4 (left) and CD8 (right) T cell activation in response to Spike peptides in patients (after D2) vs. HD after D2 and D3. IQR [0.02%–0.20%] for Spike-specific CD4 T cells and IQR [0.09%–1%] for Spike-specific CD8 T cells in responder RA. Mann–Whitney two-tailed *P*-value with ** for *p* < 0.01 and *** for *p* < 0.001. **(B)** Functionality of SARS-CoV-2 specific T cells after D4-induced seroconversion. Frequency of Spike-specific T cells before (D3m1) and after (D4m1) serological response in RA. CD4 (left) and CD8 (right) T cell activation in response to Spike peptides in non-responder (NR) vs. responder (R) patients. IQR of Spike-specific CD4 T cell response for D3m1 [0.005%–0.095%] and IQR for D4m1 [0.0075%–0.15%]) for CD4 and IQR of Spike-specific CD8 T cell response for D3m1 [0.005%–0.095%] vs. D4m1 [0.005%–0.14%]. Wilcoxon matched-pairs signed-rank test; *P*-value non-significant. **(C)** Detection and quantification of Spike-specific CD8 T cells in RA; the dot plot shows pre-/post-vaccination examples. MHC-Class I restricted dextramers and tetramers were used to identify *ex vivo* Spike-, CMV-, EBV-, and FLU-specific CD8 T cells. Left flow plots: example of the patient before vaccination and 1 month after D2, CMV: HLA-binding T cells (upper left region) vs. Spike: HLA-binding T cells (lower right region). Right scatterplot: frequency of T cells binding peptide: HLA after D2 in patients vs. controls. IQR [0.115%–0.81%], *n* = 61 for RA and IQR [0.08%–0.95%], *n* = 52 for HD. Mann–Whitney test; *** indicates *p* < 0.001. **(D)** Functionality of SARS-CoV-2 specific helper T cells after breakthrough infection (BTI) in patients. Frequencies of Spike-and non-Spike (M, N, O)-specific CD4 T cells are shown: red symbols, patients with PCR-documented BTI; blue dots, vaccinated only. **(E)** Quantification of Spike- and non-Spike-specific CD8 T cells in vaccinated RA after BTI. Peptide: HLA class I dextramers and tetramers were used to identify *ex vivo* Spike-, non-Spike, CMV-, EBV-, and FLU-specific CD8 T cells. Mann–Whitney test, *P*-value non-significant (see also **Supplementary Figures S4**, **S5**).

We first asked whether superior T cell responses could be related to serological responses after D3 and therefore tested Spike-specific (WT and VOC) responses in high-responders vs. non-responder RA. We found that CD4 T cell response in high-responders (median 0.095%) was not significantly different from non-responder RA (*p* = 0.34). This response was also not different from HD D2m1 (*p* = 0.15). However, CD8 T cell responses were enhanced in high-responder RA (median 0.44%) and statistically significant in comparison to non-responder RA (*p* = 0.0009) and HD D2m1 (*p* = 0.0061) (**Figure 3A**). We performed a similar approach for patients with serological response after D4 and added expression of the activation marker CD137 to improve the sensitivity of our activation assay (**Figure 3B**). The longitudinal follow-up of these patients did not suggest any clear differences (D3m1 median 0.03% vs. D4m1 median 0.07%) for CD4 and (D3m1 median 0.005% vs. D4m1 median 0.005%) for CD8 T cell responses (**Figure 3B**) (22). Moreover, T cell response, directed against the mutated peptides coding for Spike (Delta, Omicron) was preserved in patients across the time points (data not shown).

Next, we assessed whether the vaccine could induce long-lasting immunity by the characterization of the Spike-specific T cell differentiation profile. Responding Spike-specific CD4 and CD8 T cells were mostly memory cells (median 92% and 75%, respectively). Moreover, we found a significant skewing to central memory cells in both CD4 (CD45RA^-^CD27^+^, median 42.9%) and CD8 T cells (median 22.9%), although the patients also had robust effector (CD45RA^-^CD27^-^, median 42.7% for CD4 T cells and median 20% for CD8 T cells) and limited terminal effector memory responses (CD45RA^+^CD27^-^, CD4 median 7.14% and CD8 median 13.5%) (**Supplementary Figure S5A**).

### Phenotype of Spike-specific T cells after the booster vaccine dose and BTI

Next, to improve the detection sensitivity, we directly quantified and characterized Spike-specific CD8 T cells *ex vivo*. We identified the peptide HLA-multimer binding CD8 and performed a deep phenotypic analysis of Spike-, CMV-, EBV-, and Flu-specific CD8 T cells in patients. As soon as 1 month after D2, we detected a robust frequency of Spike-specific T cells (median 0.24%, *n* = 61 for 3 HLA-genotypes; see “Methods”) (**Figure 3C**; **Supplementary Figure S5B**), and these were not significantly different from HD (median 0.32%, *n* = 52) (**Figure 3C**; **Supplementary Figure S5B**). Interestingly, the frequency of T cells specific for chronic/latent viral infections was significantly increased in RA for EBV- (*p* = 0.0003, *n* = 20) and influenza- (*p* = 0.0003, *n* = 15) but not for CMV-derived peptides (*n* = 42). Spike-specific CD8 T cells had similar phenotypes as that found by mass cytometry discussed above. Cells were recently activated (expressed PD-1) and had an intermediate expression of effector molecules such as KLRG1, CD244, and GPR56 (G protein-coupled receptor 56) but with a limited expression of CD57. This signature segregated Spike-specific T cells from CMV-specific, terminally differentiated T cells (**Supplementary Figure S5C**), demonstrating that Spike-specific CD8 T cells were neither senescent nor exhausted.

Finally, the outstanding question was whether BTI could (1) recall vaccine-elicited cellular immunity against Spike-derived antigens and (2) induce new responses toward non-Spike-derived viral antigens. We found that breakthrough infections, first, boosted memory helper CD4 T cells specific for SARS-CoV-2 Spike and, second, generated *de novo* responses against non-Spike peptides (membrane, nucleocapsid, and ORF-derived peptides; **Figure 3D**). A direct *ex vivo* analysis showed that the frequencies of antigen-specific cytotoxic CD8 T cells toward Spike and non-Spike SARS-CoV-2 as well as other viral peptides from EBV, Flu, and CMV were similar between HDs and patients after BTI (**Figure 3E**). The signature of recently activated virus-specific T cells was preserved after BTI, enabling us to distinguish the immune response directed against recent challenges by SARS-CoV-2 (infection/vaccination) from persistent viral infections (CMV/EBV) (**Supplementary Figure S5D**).

## Discussion

Extensive *ex vivo* quantification, phenotyping, and *in vitro* functional analysis of vaccine-generated T cells showed normal vaccine T cell immunity in rituximab-treated patients irrespective of any observed attenuation in humoral immunity. Many rituximab-treated RA patients discontinued rituximab treatment after the first doses of the vaccine, prompting us to follow dose 4-generated B cell response as a function of time after a prolonged interruption of rituximab. Seroconversion (>2,000 BAU/mL) was significantly increased after dose 4 and was seen in 35%. RA patients treated with rituximab longer than 10 months before vaccination seroconverted and had a significantly higher RBD IgG titer than those with rituximab within 10 months. The seroconversion rate was significantly higher in patients with ACPA (IgG anti-CCP) responses and in patients with lower inflammatory scores (proteomic signature across 90 cytokines and chemokines). This persistent inflammation in patients with chronic conditions could partially impede vaccine responsiveness and enhance the severity of the disease after BTI (24, 25). Vaccination (doses 3 and 4) resulted in a newly primed and activated IgM^+^ B cell phenotype of cells expressing the memory marker CD27, the activation marker CD71, and Blimp-1, the transcription factor driving terminal B cell differentiation into plasma cells. Importantly, during BTI, the expansion of vaccine-generated B cells was sufficient to support the full maturation of cross-reactive (anti-Omicron) neutralizing anti-RBD IgG Abs. These patients also responded normally to non-Spike (i.e., non-vaccine antigens) for both T and B cells, demonstrating the development of broad immunity toward SARS-CoV-2.

A fine analysis of the inflammatory proteomics revealed that the signature associated with Th1/Th17 immunity (IL-12p40) correlated with IgG anti-CCP Abs, suggesting Th1/Th17-driven auto-immunity (26, 27) and a specific enrichment for RA-specific pathways.

To improve humoral vaccine responsiveness in RA patients, pausing rituximab is a tempting solution. However, prolonging the interval between rituximab doses in RA patients has a dilemma: it favors the seroconversion to SARS-CoV-2 mRNA vaccines, but it could also lead to the resurgence of auto-reactive B cells, secreting inflammatory autoantibodies such as pathogenic anti-CCP IgG (28–30). Conversely, high anti-CCP antibody titers predict a good response to rituximab in patients with active rheumatoid arthritis (31). However, the clinical importance of anti-CCP levels for monitoring disease activity is reduced due to the relatively stable secretion by long-lived plasma cells (that lack CD20) from the bone marrow, spleen, or syncytial tissues (32). Nevertheless, recent publications have described a positive correlation between ACPA and pathogenic citrulline-specific CD4 or CD8 T cells in the synovial fluid (33) and peripheral blood (34), suggesting a collaboration of auto-reactive B and T cells. In the current results, patients with lower inflammatory scores had higher anti-SARS-CoV-2 RBD IgG antibodies, a finding that suggests that ongoing inflammation and autoimmunity may, to some extent, supplant vaccine responses. Monitoring T and B cell autoimmunity to citrulline peptides in RA patients deserves future investigation, including the genetic characterization of patients and the identification of autoantigens (35) (36, 37). Enhanced oxidative stress in RA patients (as suggested in the current data by increased GDF-15) can also generate isoforms of vimentin with specific citrullination and mutation, reinforcing citrullinated mutated vimentin as an important autoantigen in RA (38, 39).

B cells express CD20 from the pre-B cell stage but lose this marker during plasmablast and plasma cell differentiation (40). CD20^-^ plasma cells are long-lasting (40, 41), and the serum levels of IgG antibodies against childhood vaccines such as measles and tetanus remained unchanged after rituximab therapy (42). The normal decay rate of the IgG anti-RBD after D4 in the current study suggests that enough B cells have migrated to the long-lived plasma cell niches despite the removal of CD20^+^ memory B cells and alteration of B cell reconstitution by rituximab. Moreover, rituximab-resistant B cell differentiation and plasma cell genesis have also been described in the gut (43). Similarly, a splenic niche of rituximab-resistant, quiescent autoreactive memory B cells has recently been described in immune thrombocytopenia patients (44). Importantly, such preserved memory B cells are mainly IgM^+^ (but can also be IgG^+^) and can overarch rituximab therapy by giving rise to plasma cells and further germinal center reactions (44). The current results of incomplete B cell differentiation even after dose 4 suggest a sub-threshold, delayed IgM-dominant vaccine B cell memory. This memory B cell pool was likely poised to expand during breakthrough infection that provided a dramatic boost to B cell differentiation in terms of cell numbers, more mature phenotype as well as increased IgG-anti-RBD levels.

T cells are necessary for the rapid and efficient resolution of COVID-19 (12, 13) and for viral control in settings of low antibody levels (14, 15). Our sensitive technology enabled us to accurately estimate the frequency and phenotype of vaccine-derived immuno-dominant epitope-specific T cells like those detected during a natural infection. This was applied both for functional *in vitro* responses as well as direct *ex vivo* quantification of Spike-specific multimers CD8 T cells. Spike-specific CD4 and CD8 T cells had a mainly central memory phenotype (CD45RA^-^CD27^+^) with the potential to self-renew and proliferate, allowing them to have a long-term persistence and effector profile (CD45RA^-^CD27^-^), with cytotoxic ability to lyse SARS-CoV-2 infected cells (45).

Here we observed an *ex vivo* frequency of Spike-specific T cells comparable to HDs, demonstrating that rituximab therapy has only a minor impact on T cells. The humoral response in patients was therefore not always associated with the enhanced functionality of Spike-specific T cells. In-depth phenotyping of Spike-specific T cells revealed effector profiles (intermediate expression of KLRG1, CD244, and GPR56, combined with a limited expression of CD57) associated with recent vaccination and significantly different from phenotypes seen in chronic infection. BTI-induced immunity supported a slightly superior immune reactivity against Spike than non-Spike specificities. Spike-specific CD8 had an appropriate effector memory phenotype without exhaustion or senescence markers to protect against severe COVID-19 (12, 13, 15, 16).

The primary limitation of our study lies in the heterogeneous nature of the patient group regarding treatment modalities and clinical contexts, which poses challenges in interpreting our findings in terms of providing conclusions for clinical practice. We aimed to deepen our insights into the pathophysiology of vaccine responsiveness in immunocompromised patients rather than concluding on the merits of the discontinuation of rituximab. Clinical evaluations to assess disease activity were limited, except those conducted in Nor-vaC immediately after the third vaccine dose (4). However, we can note that none of the patients experienced disease flares requiring hospital admissions during the pause in rituximab therapy. In conclusion, the risk of deterioration in rheumatoid arthritis *versus* the benefit of serological response should be considered individually for each patient.

Our results are relevant for future vaccination strategies in rituximab-treated patients. T cell immunity was not disturbed, and immediate vaccination disregarding time after rituximab will promote T cell immunity regardless of low frequencies of B cells and poor serological responses. Furthermore, older age, greater inflammation, and recent infusion of rituximab limited the seroconversion of RA patients. These factors can be circumvented by pausing rituximab medication (in the absence of flare or RA relapse), continuing csDMARDs, and recommending booster doses for the elderly who have lower responses.

Previous evidence has demonstrated that T cell memory to SARS-CoV-1 is long-lived (46), and current results suggest that rituximab-treated patients have successfully acquired normal T cell immunity both after vaccination and BTI. The development of durable antibody responses as shown here suggests that patients with paused rituximab medication can also develop long-lived plasma cells (42). We find it likely that most paused rituximab-treated patients have seroconverted (booster dose of vaccine and/or BTI) and therefore should have acquired durable protection against SARS-CoV-2, as illustrated by the low number of observed severe forms of the disease after BTI in our cohort of vaccinated RA. Further follow-up and detailed statistical analysis of vaccine efficacy against severe COVID-19 is merited.

## Data availability statement

The raw data supporting the conclusions of this article will be made available by the authors without undue reservation.

## Ethics statement

The studies involving humans were approved by the Regional Committees for Medical Research Ethics Southeast Norway, reference numbers 235424, 135924, and 204104; (clinicaltrials.gov NCT04798625). The studies were conducted in accordance with the local legislation and institutional requirements. The participants provided their written informed consent to participate in this study.

## Author contributions

HK: Conceptualization, Data curation, Formal analysis, Investigation, Methodology, Project administration, Supervision, Validation, Visualization, Writing – original draft, Writing – review & editing. IJ: Data curation, Investigation, Writing – review & editing. AA: Data curation, Formal analysis, Investigation, Writing – review & editing. IE: Formal analysis, Investigation, Writing – review & editing. TT: Formal analysis, Investigation, Writing – review & editing. LT: Formal analysis, Investigation, Writing – review & editing. KL: Data curation, Investigation, Project administration, Writing – review & editing. AT: Investigation, Writing – review & editing. SP: Investigation, Writing – review & editing. HØ: Investigation, Writing – review & editing. EH: Investigation, Writing – review & editing. JV: Investigation, Project administration, Resources, Writing – review & editing. KJ: Investigation, Project administration, Resources, Writing – review & editing. SS: Investigation, Project administration, Resources, Writing – review & editing. FL: Data curation, Formal analysis, Investigation, Methodology, Project administration, Resources, Supervision, Validation, Writing – review & editing. GG: Data curation, Investigation, Project administration, Resources, Writing – review & editing. LM: Conceptualization, Data curation, Formal analysis, Funding acquisition, Investigation, Methodology, Project administration, Resources, Supervision, Validation, Visualization, Writing – original draft, Writing – review & editing.
