# Supplementary material for: Dynamics of SARS-CoV-2 immunity after vaccination and breakthrough infection in rituximab-treated rheumatoid arthritis patients: a prospective cohort study
Source: Front Immunol. 2024 Feb 22;15:1296273. doi: 10.3389/fimmu.2024.1296273 (PMC10917913; doi:10.3389/fimmu.2024.1296273)
Supplement: Supplementary file 1 [file DataSheet_1.pdf]

Supplementary Data and Materials

Dynamics of SARS-CoV-2 immunity after vaccination and breakthrough infection in rituximab treated rheumatoid arthritis patients: a prospective, cohort study

Hassen Kared, et al

Inventory

Supplementary Methods

Supplementary Table S1

Supplementary Figure Legends

Supplementary Methods

Patients

A subgroup of rituximab patients (n=10) who received three vaccine doses later transitioned to other biological therapies (TNFi) and were consequently excluded from the ongoing analyses post-therapy switch. Throughout the study period, the majority of patients maintained stability in their co-medications. Only a small number of patients (7) underwent medication changes, including an increase in glucocorticoid dose (1), adjustments to methotrexate dose (2), discontinuation of methotrexate (3), and initiation of methotrexate (1).

Serology

The detection after D4 of total IgG, or subclasses IgG1-4, and antibodies against citrullinated proteins (ACPA) measured as anti-cyclic citrullinated peptide (anti-CCP) by the EliA technique on a Phadia instrument, Thermo Fisher Scientific, was performed at the Section of Medical Immunology, Department of Immunology and Transfusion Medicine (Oslo University Hospital). The Cut off-reference value was 10 U/mL for anti-CCP antibodies (measurement range>1 and<340).

ELISA

The plasma of patients and HD were collected before D1 and cryo-preserved. The following ELISA kits were used according to manufacturer protocols. From R&D Systems: Human CD14 DuoSet ELISA (DY383), Human CD163 DuoSet ELISA (DY1607), Human LBP DuoSet ELISA (DY870-05), Human Galectin-9 DuoSet ELISA (DY2045), Human GDF-15 Quantikine ELISA Kit (DGD150), Human CXCL4/PF4 Quantikine ELISA (Kit DPF40), Human IFN-alpha (41100); from Ebioscience: Human MPO Instant ELISA Kit (BMS2038INST); from Thermo Scientific: Invitrogen novex IP 10 Human ELISA Kit (KAC2361); from Abcam Human C-Reactive Protein/CRP (Ab99995); from MyBioSource: Human zonulin ELISA Kit (MBS706368); from Meso Scale diagnostics: human Calprotectin (F21YB-3).

Olink® Target 96 inflammatory panel

The inflammatory environment of patients, after D4 or BTI was determined by Olink Proteomic Proximity Extension Assay (PEA) technology. The Olink® Target 96 inflammatory panel was used on an Olink® Signature Q100 instrument according to the manufacturer´s instructions. The inflammation score was calculated by assigning one point for each molecule with a fold change superior to 1 in comparison to the median NPX value of HD pre-vaccine. Beta-NGF and TSLP were excluded from the analysis due to insufficient sensitivity in our assay and we added 10 to have a maximum score of 100 (range 10-100).

Flow cytometry

Thawed peripheral blood mononuclear cells (PBMCs) collected at baseline and 2-4 weeks following D2, D3, D4, or BTI in a subset of patients were stained with antibody panels (see [21]) to define the phenotype of specific T cell responses to Spike peptides and B cell responses to RBD or Spike protein as described [21]. HLA-restricted Spike-specific CD8 T cells were detected ex-vivo by Dextramers (Immudex, Copenhagen, Denmark) or Flex-T monomers (BioLegend) tetramerized with Streptavidin fluorochromes [21].
Cells were acquired on a BD FACSymphony (BD Biosciences) or Attune NxT (ThermoFisher). Frequency values were calculated based on the parent immune cell population percentage and phenotypic markers were gated individually for each sample and calculated as % of positive cells. High-dimensional phenotypic profiles and sample

distributions were shown using uniform manifold approximation and projection. Data analysis was performed using CYTOGRAPHER® (ImmunoScape cloud-based analytical software), custom R-scripts, GraphPad Prism (GraphPad Software), and FlowJo v10 software (BD Life Sciences). Statistical significance was set at a threshold of $*p < 0{\cdot}05$, $**p < 0{\cdot}01$, and $***p < 0{\cdot}001$.
PBMCs were stained with antibodies to the markers as indicated. VeriCells PBMC (BioLegend) were included as controls. The following mAbs and stains were utilized for acquisition on a BD FACSymphony: BB515 Mouse Anti-Human CD279 (PD-1) Clone EH12.1, BD Biosciences, PerCP-eFluor 710, KLRG1 Monoclonal Antibody (13F12F2), eBioscience, PE/Cyanine7 anti-human GPR56, Clone CG4, Nordic Biosite, Alexa Fluor 700 anti-human CD244 (2B4), clone C1.7, Nordic Biosite, APC/Cyanine7 anti-human HLA-DR, clone L243, Nordic Biosite, BV480 Rat Anti-Human CXCR5 (CD185) (Clone: RF8B2) BD Biosciences, BB515 Mouse Anti-Human CD38, clone , HIT2 BD Biosciences, Brilliant Violet 570™ anti-human CD3, Nordic Biosite, Brilliant Violet 605, CD127 Mouse anti Human, Clone HIL 7R M21, BD Biosciences, Brilliant Violet 650, CD161 Mouse anti Human, clone: DX12, BD Biosciences, BV711 Mouse Anti-Human TIM-3 (CD366), clone 7D3, BD Biosciences, BV750 Mouse Anti-Human CD8, clone SK1, BD Biosciences, Brilliant Violet 785™ anti-human CD57 Recombinant, clone QA17A04, Nordic Biosite, BV421 Mouse Anti-Human CD319 (CRACC), BD Biosciences, BUV395 Mouse Anti-Human TIGIT, clone 741182, BD Biosciences, Live/dead™ Fixable Blue Dead Cell Stain Kit, for UV excitation, Thermo Fisher Scientific, BUV563 Mouse Anti-Human CD45RO, clone UCHL1, BD Biosciences, BUV615 Mouse Anti-Human CD95, clone DX, BD Biosciences, BUV661 Mouse Anti-Human CD4, clone SK3, BD Biosciences, BUV737 Mouse Anti-Human CD38, clone HB7, BD Biosciences, BUV805 Mouse Anti-Human CD27, clone L128, BD Biosciences.

Functional assay - Detection of SARS-CoV-2 specific memory T cells
The functionality of Spike- and non-Spike-specific T cells was assessed in vitro by combined activation-induced markers (AIM) and intra-cellular cytokines secretion assays, as described[21]. Thawed cells were stimulated with SARS-CoV-2 PepTivator Spike protein peptides (Wuhan-Hu-1), Nucleoprotein and Membrane protein (Miltenyi Biotech), and immunodominant sequences from the whole proteome (structural and non-structural proteins) consisting of 15-mer sequences with 11 amino acid overlap covering the immunodominant parts of the corresponding proteins for 16 hours as described[21]. Cells were stained to detect $CD4^+$ and $CD8^+$ T cells and to quantify CD40L (CD154), CD137, interferon-gamma (IFNγ), interleukin-2 (IL-2), or tumor necrosis factor (TNF) expression by flow cytometry (Attune NxT, ThermoFisher).

Flow cytometry - Detection of SARS-CoV-2 specific memory CD8 T cells
Antigen-specific CD8 T cells were detected using PE-conjugated Dextramers targeting Spike and restricted to HLA-A*0101 (LTDEMIAQY), HLA-A*0201 (YLQPRTFLL), HLA-A*2402 (QYIKWPWYI), and HLA-B*0702 (SPRRARSVA). To increase the sensitivity of our assay and based on our previous publication[16], we have completed our panel of epitopes by using Flex-T tetramer according to the manufacturer´s instructions. We UV-exchanged peptides for Spike epitopes restricted to HLA-A*0101 (YTNSFTRGVY), HLA-A*0201 (LITGRLQSL, and RLNEVAKNL), HLA-A*2402 (NYNYLYRLF), and HLA-B*0702 (APHGVVFL) and tetramerized with Streptavidin-PE (Biolegend). CMV-and EBV/FLU-specific CD8 T cells were generated similarly and tetramerized using Streptavidin-PECF594 (Biolegend) and Streptavidin-PE-Cy5 respectively. CMV-derived epitopes were for HLA-

A*0101 (VTEHDTLLY), HLA-A*0201 (NLVPMVATV), HLA-A*2402 (QYDPVAALF), and HLA-B*0702 (RPHERNGFTVL) and EBV derived epitopes were for HLA-A*0101 (CTELKLSDY/FLU), HLA-A*0201 (FLYALALLL/EBV), and HLA-B*0702 (RPPIFIRRL).

The peptides that bound HLA-A*0101, HLA-A*0201, HLA-A*2402, or HLA-B*0702 Dextramer/Flex-T were derived from SARS-CoV-2 (Spike or non-Spike), CMV, EBV or Influenza virus. Note that single patients could have several or no measurable T cell specificities, depending on the HLA genotype. All peptides were ordered from Genscript with a purity above 85% by HPLC purification and mass spectrometry. Lyophilized peptides were reconstituted at a stock concentration of 10 mM in DMSO.
Cells were acquired on a BD FACSymphony (BD Biosciences), Attune NxT (ThermoFisher), or on a Helios mass cytometer (Fluidigm). Frequency values were calculated based on the parent immune cell population percentage and phenotypic markers were gated individually for each sample and calculated as % of positive cells. High-dimensional phenotypic profiles and sample distributions were shown using uniform manifold approximation and projection.

Flow cytometry - Detection of SARS-CoV-2 specific memory B cells
Spike-specific B cells were detected using either sequential staining of biotinylated Recombinant SARS-CoV-2 Spike-Trimer (HEK) (Miltenyi) combined with streptavidin-PE or with probes already conjugated with Alexa Fluor 647 for Spike RBD (R&D Systems) and conjugated with Alexa Fluor 488 for Full- length spike protein (R&D Systems), see[21,22]. $2x10^6$ cryo-preserved PBMC samples were transferred in a 96-well U-bottom plate. Cells were first stained with Fc block (BD Biosciences) for 15 minutes at room temperature. Cells were then washed and stained separately with either 100 ng of Spike Trimer alone or probe master mix containing 200ng spike-A488, and 25ng RBD-A647 for 1 hour at 4C. Following incubation with an antigen probe, cells were washed twice and stained with Blue Live Dead (Thermo Fischer) for 10 minutes at room temperature. Cells were washed again and stained with anti-CD7, anti-CD14, anti-CD19, anti-CD20, anti-CD21, anti-CD24, anti-CD27, anti-CD38, anti-CD71, anti-IgD, anti-IgM, anti-IgG, HLA-DR, and CXCR5 for 30 minutes on ice. Cells stained with the Spike Trimer were fixed with the transcription factor buffer (Thermo Fischer) and intra-cellularly stained for IRF4 and Blimp-1. Cells stained with RBD and full Spike were fixed overnight in 1 % PFA. Samples were acquired on FACSymphony. All Antibodies are purchased from BD biosciences except Blimp-1 and IRF4 (Thermo Fischer).

Mass cytometry
For in-depth analysis of spike-specific B and T cells, thawed PBMCs from the Omicron - and Delta-infected individuals were run alongside uninfected healthy vaccinated controls and VeriCellsTM PBMC (BioLegend). The following mAbs from Fluidigm were used for Helios Cytof: y89 anti-CD45, 106Cd* anti-CD45, 110Cd* anti-CD45, 111Cd anti-CD19, 112Cd anti-CD45, 114Cd anti-CD45, 116Cd anti-CD45, 141Pr anti-CCR6, 142Nd anti-CD57, 143Nd anti-CD45RA, 144Nd anti-CD38, 145Nd anti-CD4 , 146Nd anti-CD8/IgD, 147Sm anti-CD20, 148Nd anti-CD14, 149Sm anti-CD25, 150Nd anti-TCRVa7.2, 151Eu anti-lambda, 152Sm anti-TCRgd, 153Eu anti-TIM-3, 154Sm anti-CD3, 155Gd anti-CD27, 156Gd anti-CXCR3, 158Gd100 anti-CCR4, 159Tb anti-TIGIT, 160Gd anti-Kappa, 161Dy anti-CD160, 162Dy anti-CD95, 163Dy anti-CRTH2, 164Dy anti-CD161, 165Ho anti-CD127, 166Er anti-CD85j, 167Er anti-CCR7, 168Er anti-CD71, 169Tm anti-NKG2A, 170Er anti-HLA-DR, 171Yb anti-CXCR5, 172Yb anti-KLRG1, 173Yb anti-CD141, 174Yb anti-PD anti-1/CD279, 176Yb anti-CD56, 209Bi anti-CD16, 194Pt Cisplatin live/dead. Samples were collected on a Helios mass cytometer (Fluidigm), with samples resuspended with 20% equilibration beads

spiked into each sample to allow for signal normalization. Samples were normalized using the Helios software.

Supplementary Table S1. Baseline characteristics cellular sub-analyses

	**B cell analyses**<br><br>**N=19**	**T cell analyses**<br><br>**N=30**	**Dextramers**<br><br>**N=13**	**Inflammation markers**<br><br>**N=10**	**BTI cellular analyses**<br><br>**N=4**
**Age, median (IQR)**	59·0 (47·0–66·0)	60·0 (54·0–67·0)	61·0 (56·0–68·0)	67·0 (60·0–70·0)	67·0 (53·5–75·5)
**Female sex, no (%)**	16 (84)	26 (87)	11 (85)	8 (80)	4 (100)
**Rituximab monotherapy, no (%)**	5 (26)	8 (27)	2 (15)	2 (20)	0
**Comedication**					
Methotrexate	10 (53)	15 (50)	7 (54)	7 (70)	3 (75)
Prednisolone	1 (5)	1 (3)	0	1 (10)	1 (25)
Sulfasalazine	0	2 (7)	2 (15)	0	0
Leflunomide	2 (11)	3 (10)	1 (8)	0	0
Plaquenil	1 (5)	1 (3)	1 (8)	0	0
**Time on rituxumab treatment before D1, median years (IQR)**	4·9 (1·7–9·3)	4·8 (2·7–9·2)	8·3 (4·4–9·3)	7·7 (2·7–8·9)	6·5 (3·1–8·8)
**Number of rituximab infusions before D1, median (IQR)**	9·0 (3·0–18·0)	9·0 (5·0–18·0)	16·0 (9·0–18·0)	14·0 (3·0–17·0)	12·5 (6·0–17·0)
**Time from last rituximab infusion to event, days (IQR)**					
Dose 2	193 (122–258)	150 (121–239)	122 (112–127)	122 (81–193)	148 (97–188)
Dose 3	183 (123–243)	180 (124–226)	163 (132–184)	174 (154–218)	139 (128–147)
Dose 4	255 (148–371)	284 (144–363)	286 (118–332)	230 (113–359)	100 (60–213)
BTI	212 (122–382)	212 (126–370)	328 (168–382)	337 (195–466)	168 (136–206)
**Vaccine types, n (%)**					
**Dose 1 and 2**					
mRNA-1273	5 (26)	9 (30)	3 (23)	2 (20)	1 (25)
BNT162b2	14 (74)	21 (70)	10 (77)	8 (80)	3 (75)
**Dose 3**					
mRNA-1273	7 (37)	11 (37)	3 (23)	3 (30)	1 (25)
BNT162b2	12 (63)	19 (63)	10 (77)	7 (70)	3 (75)
**Dose 4**					
mRNA-1273	8 (44)	13 (48)	7 (59)	8 (80)	2 (50)
BNT162b2	8 (44)	12 (44)	4 (33)	2 (20)	2 (50)
CBA01	1 (6)	1 (4)	1 (8)	0	0
CBA45	1 (6)	1 (4)	0	0	0
**BTI the whole period, n (%)**	15 (79)	21 (70)	8 (62)	6 (60)	4 (100)
**Time from last vaccine to BTI, median days (IQR)**	100 (39–193)	100 (48–169)	67 (52–101)	86 (60–103)	72 (63–86)
BTI= breakthrough infection. D1= vaccine dose 1. IQR= interquartile range.					

Supplementary Figure Legends

Supplementary Figure S1. Serological characteristics of rituximab treated RA during BTI.

A. Overview of study. Rituximab-treated RA patients were followed 1 month after successive vaccinations and after SARS-CoV-2 breakthrough infection. PBMCs were evaluated with mass cytometry and advanced flow cytometry to quantify vaccine responder cells, this included detection and phenotyping of vaccine-generated B cells in seroconverted patients. Specific T cells were detected directly ex vivo with peptide: HLA class I multimers and were phenotyped in terms of expression for activation, exhaustion, and effector markers. Further, in vitro stimulation assays with SARS-CoV-2 peptides or controls allowed quantification of polyfunctional responses as measured by expression of surface markers (CD154, CD137), cytokines (IL-2, IFN-γ, TNF) and cytotoxicity proteins (Perforin, Granzyme B).

B. Serological response of RA patients during vaccination. IgG anti-RBD Spike SARS-CoV-2 response in rituximab treated RA (n=65, dose 2 (D2); n=63, D3; n=56, D4) vs. HD (n=167, D2; n=168, D3) one month after vaccination. The median titer at D3 for HD was 8180 BAU/mL [IQR 6348-11545]. Scatter plots with medians and interquartile ranges are shown. Dotted lines demarcate ranges (>2000, 200-2000, 20-200 BAU/mL), numbers of individuals in each range are shown. Mann Whitney tests with two-tailed P values are p<0.0001 for D2m1 and D3m1.

C. Biplot showing anti-RBD responses as a function of time between rituximab infusion and D4. The dotted vertical line represents the median interval time used to segregate patients (10 months). Linear regression is shown. Pearson correlation coefficient and significance are indicated.

D. Overview of serological response according to anti-citrullinated protein auto-antibodies. The Total IgG and subclass IgG2 concentrations are represented in scatter plots in RA patients at D4. The range of total IgG values was in HDs [IQR 6.1-15.87], ACPA-positive RA [IQR 6.75-8.94], and ACPA-negative RA [IQR 5.69-8.07]). The range of IgG2 values was IQR 1.63-2.39 and IQR 1.3-1.87 for ACPA-positive and negative RA respectively). The cut-off for ACPA was 10 U/mL. Mann Whitney test, two-tailed P values are indicated.

E. Time-evolution of serological response. XY plots show anti-SARS-CoV-2 RBD titer trajectories with time (days), up: RA after D2/D3/D4, lower left: HD after D4, lower right: anti-TNF-treated RA after D3. Slopes corresponding to $T_{1/2}$ of 60 days are shown in HD and RA, and $T_{1/2}$ of 60 days, 30 days, and 15 days for anti-TNF-treated RA as indicative values.

F. Decay of serological response Violin plots show the distribution of Ab decay in RA (n=12/17/14 after D2/D3/D4 respectively), healthy controls (N=54), and RA treated with TNF (N=81) that achieved anti-SARS-CoV-2 RBD >2000 BAU/mL (>1000 BAU/mL for RA D2/D3). Slopes were calculated by linear regression with [median -13 $BAUmL^{-1}d^{-1}$, IQR -19 to -11 $BAUmL^{-1}d^{-1}$] in D2 RA, [median -10 $BAUmL^{-1}d^{-1}$, IQR -22 to -6 $BAUmL^{-1}d^{-1}$] in D3 RA, [median -36 $BAUmL^{-1}d^{-1}$, IQR -72 to -16 $BAUmL^{-1}d^{-1}$] in D4 RA, vs. [median -39 $BAUmL^{-1}d^{-1}$, IQR -130 to -29 $BAUmL^{-1}d^{-1}$] in D3 HD, and [median -65 $BAUmL^{-1}d^{-1}$, IQR -91 to -44 $BAUmL^{-1}d^{-1}$] in D3 TNFi-RA, Mann Whitney test, two-tailed P values are indicated.

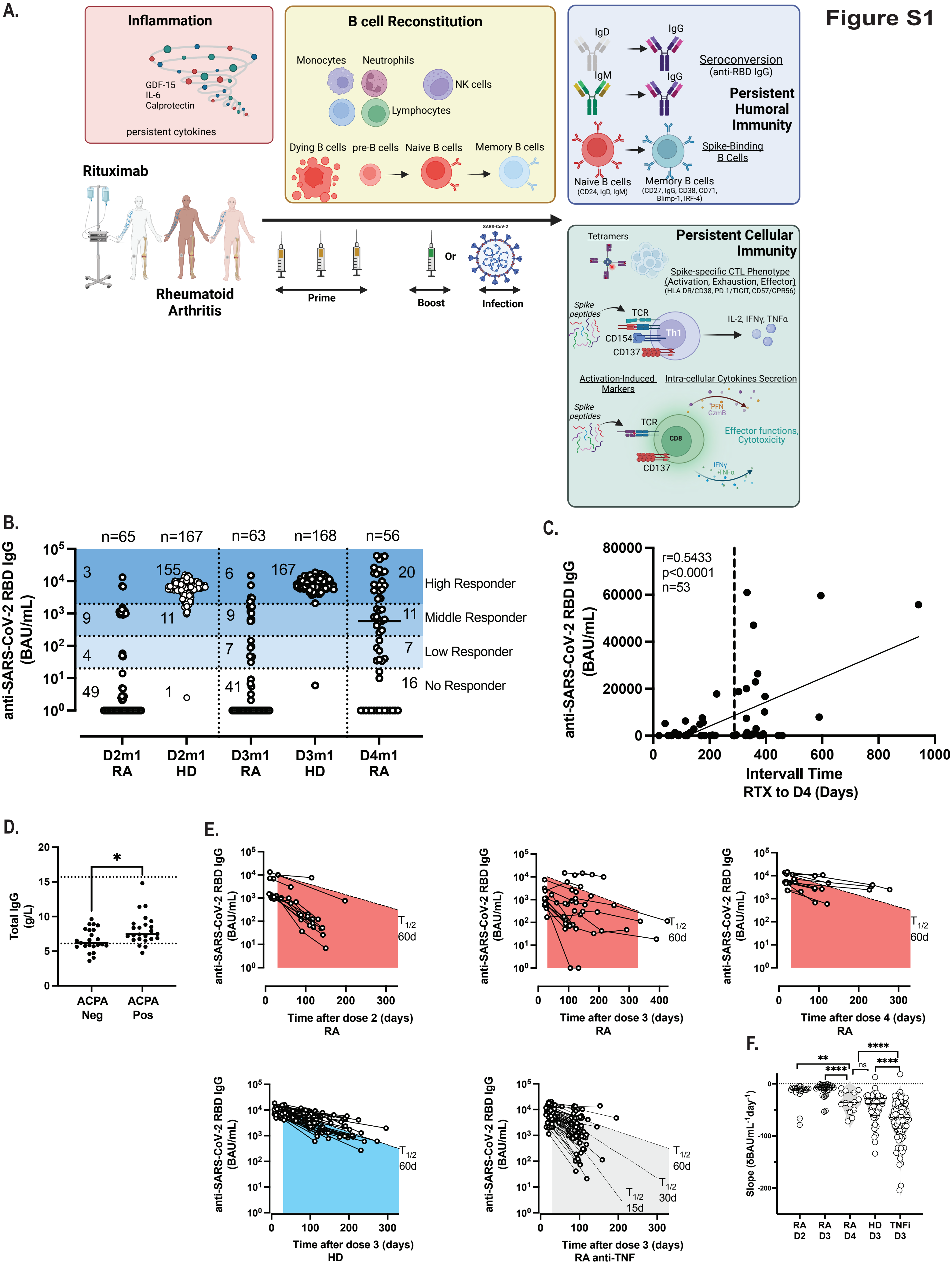

Figure S1
A.
Inflammation
GDF-15
IL-6
Calprotectin
persistent cytokines
B cell Reconstitution
Monocytes
Neutrophils
NK cells
Lymphocytes
Dying B cells
pre-B cells
Naive B cells
Memory B cells
IgD
IgG
IgM
IgG
Seroconversion
(anti-RBD IgG)
Persistent Humoral Immunity
Spike-Binding B Cells
Naive B cells
(CD24, IgD, IgM)
Memory B cells
(CD27, IgG, CD38, CD71, Blimp-1, IRF-4)
Rituximab
Rheumatoid Arthritis
Prime
Boost
Or
SARS-CoV-2
Infection
Tetramers
Persistent Cellular Immunity
Spike-specific CTL Phenotype
(Activation, Exhaustion, Effector)
(HLA-DR/CD38, PD-1/TIGIT, CD57/GPR56)
Spike peptides
TCR
Th1
CD154
CD137
IL-2, IFNγ, TNFα
Activation-Induced Markers
Intra-cellular Cytokines Secretion
PFN
GzmB
CD8
Effector functions, Cytotoxicity
IFNγ
TNFα
B.
n=65
n=167
n=63
n=168
n=56
anti-SARS-CoV-2 RBD IgG (BAU/mL)
High Responder
Middle Responder
Low Responder
No Responder
D2m1 RA
D2m1 HD
D3m1 RA
D3m1 HD
D4m1 RA
C.
r=0.5433
p<0.0001
n=53
anti-SARS-CoV-2 RBD IgG (BAU/mL)
Intervall Time
RTX to D4 (Days)
D.
Total IgG (g/L)
ACPA Neg
ACPA Pos
E.
Time after dose 2 (days)
RA
Time after dose 3 (days)
RA
Time after dose 4 (days)
RA
Time after dose 3 (days)
HD
Time after dose 3 (days)
RA anti-TNF
T1/2 60d
T1/2 30d
T1/2 15d
F.
Slope (δBAUmL-1day-1)
RA D2
RA D3
RA D4
HD D3
TNFi D3
ns

Supplementary Figure S2. The inflammatory status of rituximab-treated RA patients

A. Baseline quantification of pro-inflammatory molecules. Pre-vaccination sera were measured by Elisa for the indicated markers. Significant differences for GDF-15 [IQR 644-1078 pg/mL], Calprotectin [IQR 29500-44600 pg/mL], and IL-6 [IQR 12·6-14·9 pg/mL] were evaluated by a Mann-Whitney Test two-tailed, * and **** denote p<0·05, and p<0.0001.
B. Specific inflammatory signature in pre-vaccinated RA patients. The correlogram described the significant correlation between the different pro-inflammatory molecules. Pearson correlations with FDR are indicated with * for p<0.05 and ** for p<0.01.
C. Inflammatory proteomic profile of RA patients after vaccination (D4) or breakthrough infection. Plasma concentration was measured by Olink technology. Normalized protein expression (NPX) is represented for HD and RA by a scaled cold-to-hot heatmap.
D. Inflammatory signature of RA patients after vaccination (D4) or breakthrough infection. Visualization by Principal Component Analysis of inflammatory molecule in HD and RA. Ellipses were automatically generated to visualize the distribution of each patient group.
E. Quantification of inflammatory signature in RA patients after vaccination (D4) or breakthrough infection. Fold change and significance between the protein's expression in different patient groups and controls were represented in volcano plots.
F. Inflammation and clinical parameters. The additive inflammatory score (see Methods) was calculated for each patient and RA D4 patients were segregated according to vaccine serostatus, age, and interval time since the last rituximab infusion. Mann-Whitney Test two-tailed, *, ** and *** denote p<0.05, p<0.01 and p<0.001.
G. Inflammation and auto-immunity. The concentration of anti-CCP IgG autoantibodies or total IgG and the protein expression of IL-12p40 were measured at D4 in RA. Pearson correlations are indicated with * for p=0.034 and ** for p=0.002.
H. KEGG pathway analysis of inflammatory signature. The proteins constituting the inflammatory score for each group of patients were analyzed to identify and quantify the enriched networks of interacting molecules and functions.

Figure S2

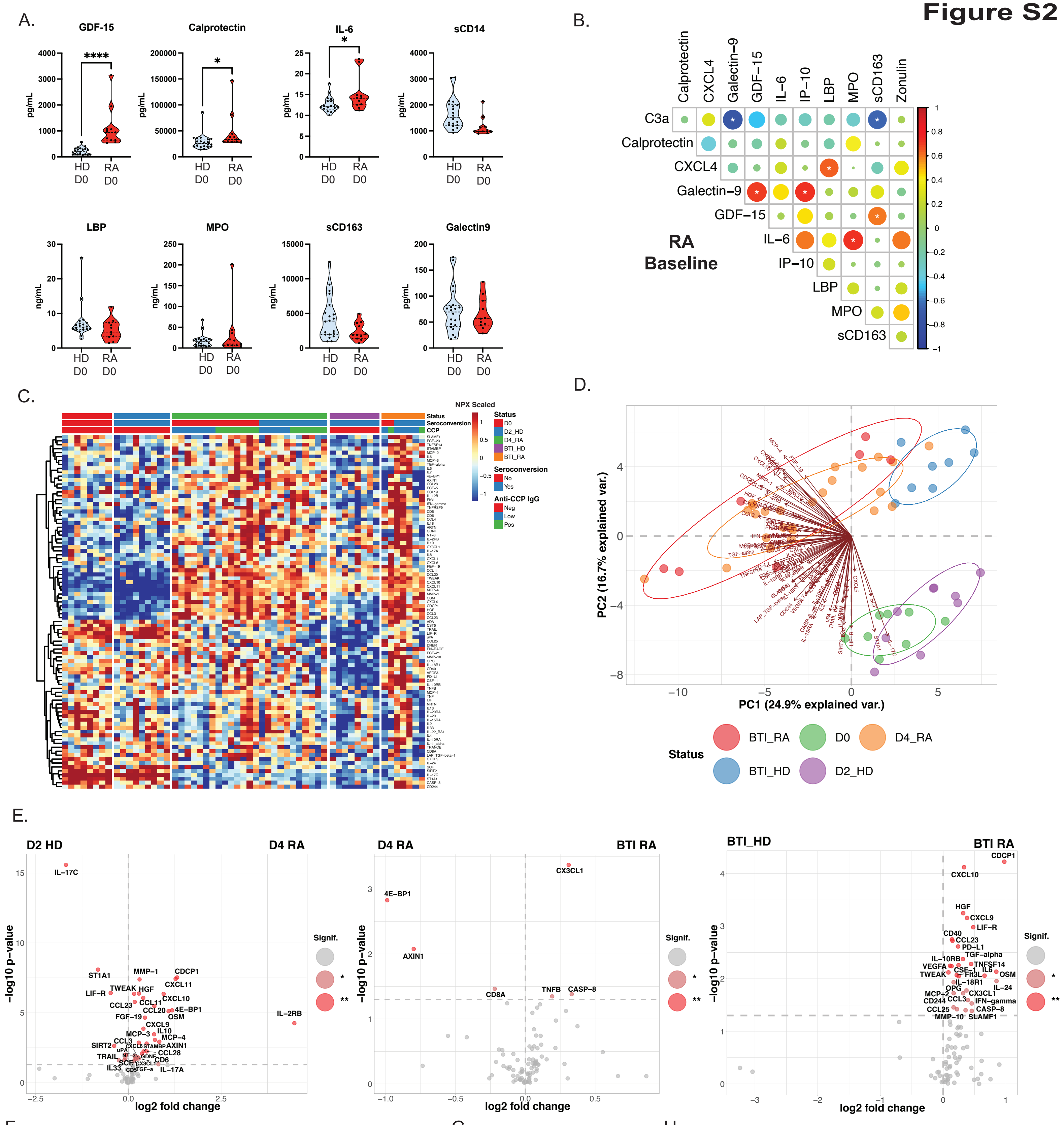

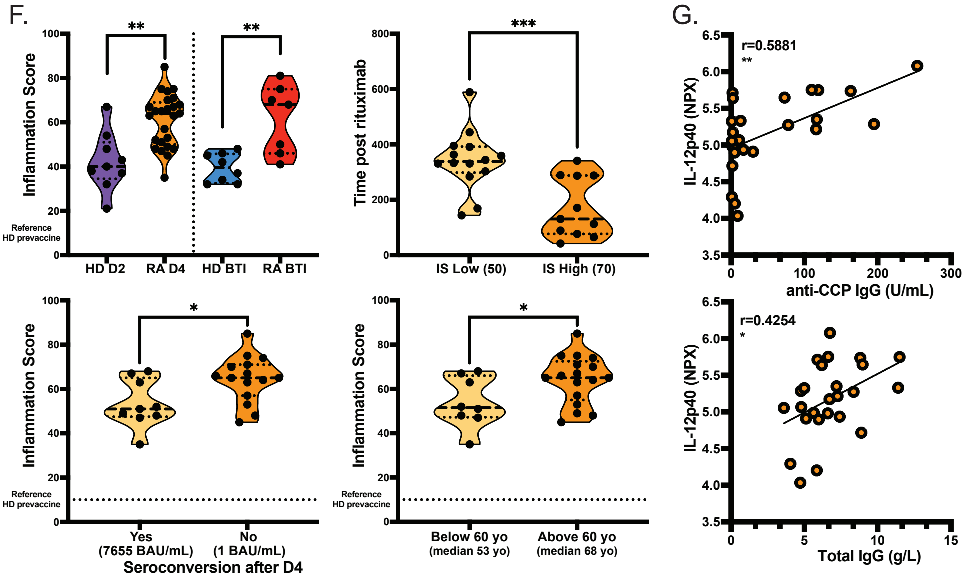

H.

patients	term description	strength	FDR	#term ID
RA D4<br>RA BTI<br>HD BTI<br>HD D2	Cytokine-cytokine receptor interaction	1.61<br>1.64<br>1.59<br>1.64	4.54E-36<br>1.30E-41<br>1.53E-17<br>1.16E-12	hsa 04060
RA D4<br>RA BTI<br>HD BTI<br>HD D2	Viral protein interaction with cytokine and cytokine receptor	1.95<br>2<br>1.99<br>2.01	9.60E-33<br>6.14E-39<br>6.91E-19<br>1.16E-12	hsa 04061
RA D4<br>RA BTI<br>HD BTI	Chemokine signaling pathway	1.49<br>1.51<br>1.53	3.31E-15<br>1.11E-16<br>8.39E-09	hsa 04062
RA D4<br>RA BTI<br>HD BTI<br>HD D2	Rheumatoid arthritis	1.72<br>1.67<br>1.44<br>1.76	3.50E-14<br>2.58E-12<br>7.90E-03<br>7.79E-05	hsa 05323
RA D4<br>RA BTI<br>HD BTI<br>HD D2	IL-17 signaling pathway	1.65<br>1.59<br>1.53	3.43E-12<br>2.06E-10<br>5.30E-04	hsa 04657

Supplementary Figure S3. Phenotype of B cells after vaccination and BTI
A. Phenotype of B cells from seroconverted patients. Representative flow plots from 3 patients (1,2,3) after D2 or D3 and 4 HD (4, 5, 6, 7) after D2. Dot plots show IgD vs. CD27, IgD vs CD38, IgD vs IgM, IgD vs λ L chain, and IgD vs κ L chain.
B. Heterogeneity of B cells after successful vaccination in RA patients. Unsupervised analysis of B cells from seroconverted patients, tSNE visualization of 6 clusters automatically identified by phenograph is shown.
C. Identification of B cell clusters. Clusters 1-6 from B are presented in a clustered heat map for the indicated markers
D. Distribution of B cell frequencies in clusters 1-6 divided into paired seroconverted after 3rd dose vs non-seroconverted patients after 2nd dose.
E. Reconstitution of peripheral B cells in rituximab-treated B cells and seroconversion. Percent live B cells of total PBMC vs IgG anti-RBD in seroconverted (red symbols) vs non-seroconverted (black).
F. Identification of WT and Omicron RBD or Spike-binding B cells after BTI. Representative examples of two HD (left) and two RA (right) showing the distribution of Spike and RBD binding B cells – B cells that bind WT or Omicron (BA.1.1) variants of Spike or RBD. See also Figure 2 D, E.

Figure S3

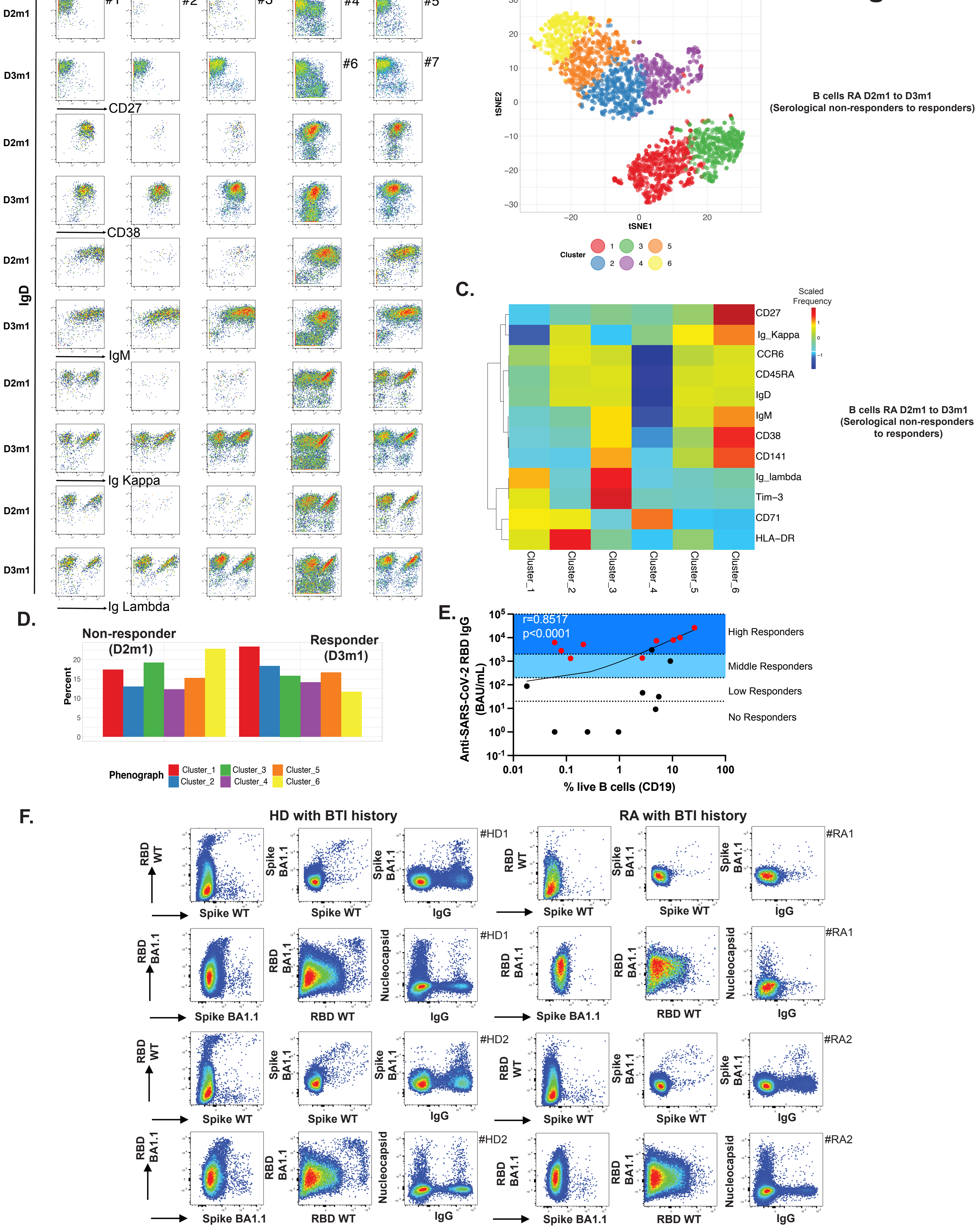

A.
RA D2m1/D3m1
HD D2m1
#1
#2
#3
#4
#5
#6
#7
D2m1
D3m1
CD27
CD38
IgM
Ig Kappa
Ig Lambda
IgD
B.
tSNE – Phenograph
tSNE2
tSNE1
Cluster
B cells RA D2m1 to D3m1
(Serological non-responders to responders)
C.
Scaled Frequency
CD27
Ig_Kappa
CCR6
CD45RA
IgD
IgM
CD38
CD141
Ig_lambda
Tim−3
CD71
HLA−DR
Cluster_1
Cluster_2
Cluster_3
Cluster_4
Cluster_5
Cluster_6
B cells RA D2m1 to D3m1
(Serological non-responders to responders)
D.
Non-responder (D2m1)
Responder (D3m1)
Percent
Phenograph
Cluster_1
Cluster_2
Cluster_3
Cluster_4
Cluster_5
Cluster_6
E.
Anti-SARS-CoV-2 RBD IgG (BAU/mL)
r=0.8517
p<0.0001
High Responders
Middle Responders
Low Responders
No Responders
% live B cells (CD19)
F.
HD with BTI history
RA with BTI history
#HD1
#HD2
#RA1
#RA2
RBD WT
RBD BA1.1
Spike BA1.1
Spike WT
Nucleocapsid
IgG

Supplementary Figure S4. Dynamics of Immune Cells during SARS-CoV-2 vaccination.
A. Visualization of immune cells during SARS-CoV-2 vaccination. Blood from patients (n=4) and healthy donors (HD, n=2) were collected longitudinally before and after vaccination. Cryo-preserved PBMCs were analyzed by CyTOF and the phenotype of live CD45 cells was visualized by a UMAP representation. The intensity expression of each marker is represented by a cold-to-hot heatmap. Clusters were automatically identified by Phenograph and FlowSOM.
B. Identification of immune cells after vaccination in patients and HD. FlowSOM-identified clusters (from A) were characterized by a scaled intensity for each marker and represented on a heatmap. Markers were automatically clustered according to their co-expression.
C. Modulation of immune cells during vaccination. Quantification of the immune cell clusters before (blue) and after SARS-CoV-2 vaccination (red).
D. Modulation of T cells during vaccination. Quantification of the immune cell subsets before (blue) and after SARS-CoV-2 vaccination (red). Naïve T cells, Memory, and Effector were identified by CyTOF as $CD45RA^{+}CCR7^{+}CD27^{+}CD95^{-/+}$, $CD45RA^{-}CD27^{+}CD95^{+,}$ and $CD45RA^{-/+}CCR7^{-}CD27^{-}CD95^{+}$ respectively.
E. Modulation of long-term memory markers in total CD8 T cells. The statistical analysis was performed by Wilcoxon signed rank test and significant differences labeled with * and ** for $p<0.05$, and $p<0.01$ respectively.

Figure S4

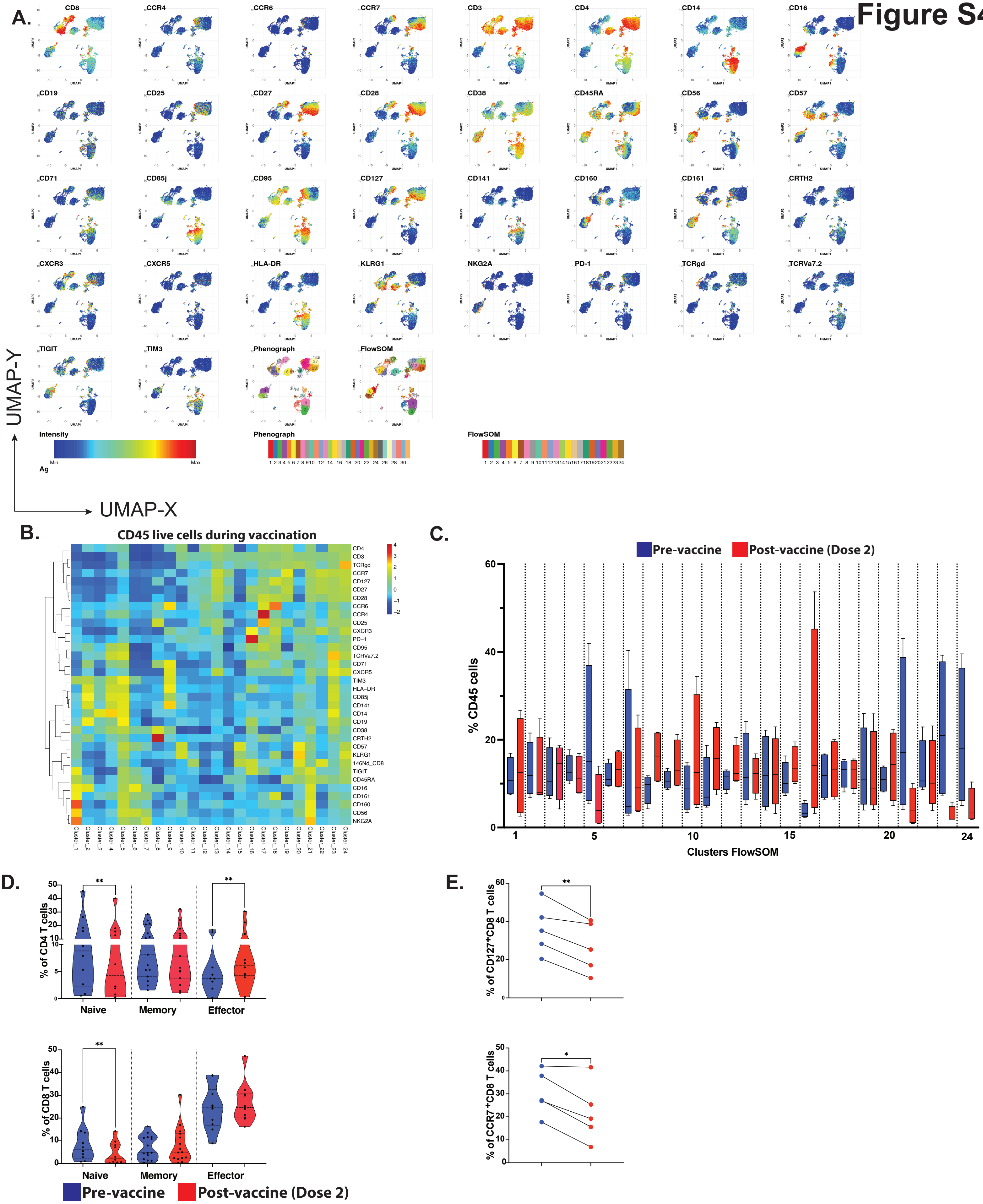

A.
CD8
CCR4
CCR6
CCR7
CD3
CD4
CD14
CD16
CD19
CD25
CD27
CD28
CD38
CD45RA
CD56
CD57
CD71
CD85j
CD95
CD127
CD141
CD160
CD161
CRTH2
CXCR3
CXCR5
HLA-DR
KLRG1
NKG2A
PD-1
TCRgd
TCRVa7.2
TIGIT
TIM3
Phenograph
FlowSOM
UMAP-Y
UMAP-X
Intensity
Min
Max
Ag
Phenograph
FlowSOM
B.
CD45 live cells during vaccination
CD4
CD3
TCRgd
CCR7
CD127
CD27
CD28
CCR6
CCR4
CD25
CXCR3
PD-1
CD95
TCRVa7.2
CD71
CXCR5
TIM3
HLA-DR
CD85j
CD141
CD14
CD19
CD38
CRTH2
CD57
KLRG1
146Nd_CD8
TIGIT
CD45RA
CD16
CD161
CD160
CD56
NKG2A
Cluster_1 … Cluster_24
C.
Pre-vaccine
Post-vaccine (Dose 2)
% CD45 cells
Clusters FlowSOM
D.
% of CD4 T cells
Naive
Memory
Effector
% of CD8 T cells
Naive
Memory
Effector
Pre-vaccine
Post-vaccine (Dose 2)
E.
% of CD127+CD8 T cells
% of CCR7+CD8 T cells

Supplementary Figure S5. Phenotype of SARS-CoV-2-specific T cells during vaccination and BTI.

A. T cell Differentiation of SARS-CoV-2-specific T cells during vaccination. PBMC from HD (blue) or patients (red) were stimulated by WT or mutated (green or white for HD and RA respectively) Spike peptides for 18h. Spike-specific $CD4^+$ cells or $CD8^+$ T cells were classified as indicated: Naïve/T_{SCM} ($CD45RA^+CD27^+$), Central Memory ($CD45RA^-CD27^+$), Effector memory ($CD45RA^-CD27^-$) or Terminal Effector ($CD45RA^+CD27^-$). Responding Spike-specific CD4 and CD8 T cells were memory IQR [87%-99%], and [67.8%-80%] respectively, including central memory cells for CD4 IQR [13.3%-83.9%]) and CD8 T cells IQR [14.5%-50·6%], effector IQR [11.8%-61.6%], and [13%-22.9%] for CD4 and CD8 T cells respectively, and terminal effector memory responses IQR [0%-15.3%] for CD4 and [5.75%-28.3%] for CD8.

B. Ex-vivo identification and phenotype of peptide: HLA multimer-specific CD8 T cells after D2. Spike-derived peptide: HLA multimer-specific T cells vs. another virus-derived peptide: HLA multimers or vs KLRG1, GPR56, or PD-1. Representative donors with different HLA specificities are shown.

C. *Ex vivo* immune phenotype of peptide: HLA multimer-specific CD8 T cells after vaccination. A cold-to-hot heatmap represents the scaled frequency of each marker expressed by antigen-specific CD8 T cells. The distribution of markers and patients was automatically performed by unsupervised hierarchical clustering. Target virus and HLA type are indicated in the top two rows (CMV, EBV, FLU, Spike, and HLA Class I subtypes). The identification of marker types as subsets (Activation, Effector, Exhaustion, Memory) is indicated in the leftmost column.

D. Analysis as in C of controls and patients after BTI.

Figure S5

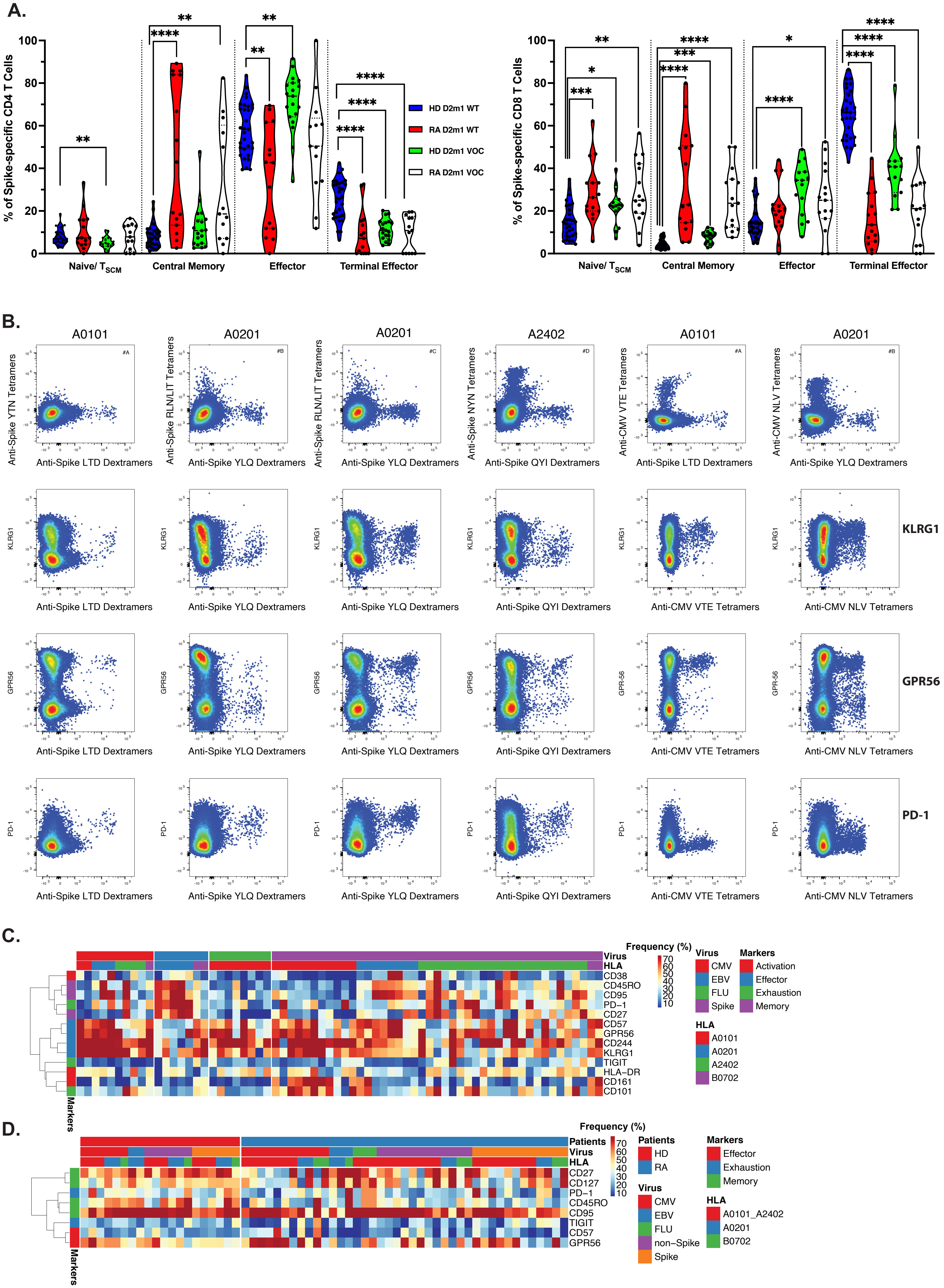
A.
% of Spike-specific CD4 T Cells
% of Spike-specific CD8 T Cells
Naive/ TSCM
Central Memory
Effector
Terminal Effector
HD D2m1 WT
RA D2m1 WT
HD D2m1 VOC
RA D2m1 VOC
B.
A0101
A0201
A0201
A2402
A0101
A0201
Anti-Spike YTN Tetramers
Anti-Spike RLN/LIT Tetramers
Anti-Spike NYN Tetramers
Anti-CMV VTE Tetramers
Anti-CMV NLV Tetramers
Anti-Spike LTD Dextramers
Anti-Spike YLQ Dextramers
Anti-Spike QYI Dextramers
KLRG1
GPR56
PD-1
C.
Frequency (%)
Virus
HLA
CD38
CD45RO
CD95
PD−1
CD27
CD57
GPR56
CD244
KLRG1
TIGIT
HLA−DR
CD161
CD101
Markers
Virus
CMV
EBV
FLU
Spike
Markers
Activation
Effector
Exhaustion
Memory
HLA
A0101
A0201
A2402
B0702
D.
Frequency (%)
Patients
Virus
HLA
CD27
CD127
PD−1
CD45RO
CD95
TIGIT
CD57
GPR56
Markers
Patients
HD
RA
Virus
CMV
EBV
FLU
non−Spike
Spike
Markers
Effector
Exhaustion
Memory
HLA
A0101_A2402
A0201
B0702
